# UPF1 contributes to the maintenance of endometrial cancer stem cell phenotype by stabilizing LINC00963

**DOI:** 10.1038/s41419-022-04707-x

**Published:** 2022-03-22

**Authors:** Hao Chen, Jian Ma, Fanfei Kong, Ning Song, Cuicui Wang, Xiaoxin Ma

**Affiliations:** 1 Department of Obstetrics and Gynecology, Shengjing Hospital of China Medical University, Shenyang, 110004 Liaoning People’s Republic of China; 2 Key Laboratory of Maternal-Fetal Medicine of Liaoning Province, Shengjing Hospital of China Medical University, Shenyang, 110004 Liaoning People’s Republic of China; 3 Key Laboratory of Obstetrics and Gynecology of Higher Education of Liaoning Province, Shengjing Hospital of China Medical University, Shenyang, 110004 Liaoning People’s Republic of China

**Keywords:** Endometrial cancer, Cancer stem cells

## Abstract

Endometrial cancer stem cells (ECSCs) play a vital role in endometrial cancer (EC) metastasis, relapse, and chemoresistance. However, the molecular mechanisms that sustain ECSCs remain elusive. Here, we showed that the expression of UPF1 was upregulated in EC tissues and ECSCs and correlated with poor clinicopathological characteristics. UPF1 silencing suppressed ECSC hallmarks, such as sphere formation ability, carboplatin resistance, migration and invasion, and cell cycle progression. UPF1 regulated the behavior and fate of ECSCs by stabilizing LINC00963. LINC00963 further shares the same miRNA response element with the core transcription factor SOX2 and relieved the suppression of SOX2 by miR-508-5p in self-renewing ECSCs. Notably, inhibition of UPF1 and LINC00963 in combination severely impaired the in vivo tumorigenic potential of ECSCs. We demonstrate that the UPF1/LINC00963/miR-508-5p/SOX2 axis has potential value in modulating ECSC maintenance, chemoresistance, and tumorigenesis in EC, which highlights a novel promising target for EC treatment.

## Introduction

Endometrial cancer (EC) originates from oncogenesis of the regenerating uterine endometrium, and it is the second-most frequent malignancy in women worldwide, with an estimated 417,367 new cases and 97,370 deaths in 2020 [1–4]. Most patients are diagnosed with early-stage disease (stage I or II), which is largely curable with surgery, occasionally combined with adjuvant therapy [5]. However, the prognosis for metastatic and advanced disease (stage III or IV) is unfavorable, with an overall 5-year survival rate of 15 to 17%, and the treatment of these patients is an unmet need [6]. To overcome this obstacle, cancer stem cells (CSCs) have received increasing attention as a promising target.

CSCs are a small subpopulation of cells that are distinguished by unlimited self-renewal and pluripotency and cause tumor growth, metastasis, recurrence, and drug resistance [7–9]. The first evidence of CSCs in ECs was derived from Hubbard et al. in 2009. Endometrial CSCs (ECSCs) likely cope with the presence of therapeutics and may be responsible for treatment failure [10]. To eliminate CSCs, further exploration is warranted to address the stemness regulation of ECSCs.

Up-frameshift mutant 1 (UPF1) is the core protein of the nonsense-mediated mRNA degradation pathway, which is tightly associated with the tumorigenesis and progression of many cancers, such as gastric [11], colorectal [12], and lung cancer [13]. Previous studies demonstrated that UPF1 was crucial for facilitating neural stem cell maintenance and proliferation [14]. UPF1 destabilized the nonsense-mediated decay (NMD) substrate encoding the TGF-β inhibitor SMAD7 and was downregulated to permit neural differentiation. UPF1 also acts as an RNA-binding protein (RBP) and plays an important role in posttranscriptional gene regulation [15, 16]. RBPs are involved in various aspects of RNA metabolism by affecting stability, splicing, RNA folding, transport, and translation [17, 18]. RNAs and RBPs bind to each other to form complexes that influence the development and progression of many diseases, including cancer [19, 20]. Previous studies demonstrated the relationship between UPF1 and lncRNAs, such as ZFPM2-AS1 [15], Linc-00313 [16], and MALAT1 [11] in many types of tumors. RNA immunoprecipitation‑sequencing (RIP‑seq) revealed that UPF1 had binding sites with LINC00963, which suggested that UPF1 functioned via LINC00963 in ECSCs. The association of UPF1 with LINC00963 has not been previously investigated.

Long noncoding RNAs (lncRNAs) modulate malignant phenotypes and CSC characteristics via epigenetic, transcriptional, and posttranscriptional regulation [21–23]. A newly identified lncRNA, LINC00963, was reported as upregulated in oral CSCs recently [24]. Suppression of LINC00963 inhibited the self-renewal, migration, and invasion of oral CSCs by reducing the expression of the multidrug-resistance transporter ABCB5. Therefore, knockdown of LINC00963 may be beneficial in the treatment of oral cancer. However, the functional significance and underlying mechanisms of LINC00963 in ECSCs remain uncharted.

LncRNAs also function as competing endogenous RNAs (ceRNAs) to regulate the targets of microRNAs (miRNAs) [25–27]. The bioinformatics software starBase (http://starbase.sysu.edu.cn/) predicted that LINC00963 targeted miR-508-5p. A previous study demonstrated the suppressive effects of miR-508-5p on the odontogenetic differentiation of human dental pulp stem cells by targeting GPNMB [28]. Sex-determining region Y-box 2 (SOX2) is a core stem cell transcription factor (SCTF) that regulates induced pluripotency and stemness [29, 30]. Overexpression of SOX2 significantly correlated with advanced histological grade and poor prognosis in EC and aggressive behaviors in ECSCs [31, 32]. StarBase analysis showed that miR-508-5p had putative binding sites with SOX2. To date, there are no reports on the functions of miR-508-5p targeting SOX2 in ECSCs.

The present study profiled the expression of UPF1, LINC00963, miR-508-5p, and SOX2 in tissues and ECSCs. We further investigated the regulatory relationships of these factors. Our results indicated that UPF1 contributed to carcinogenesis by stabilizing LINC00963, which enhanced the level of SOX2 by negatively regulating miR-508-5p to subsequently modulate self-renewal, chemoresistance, and other biological behaviors in ECSCs. Our work provides insights into the stemness regulatory mechanism in ECSCs to identify new therapeutic targets for EC.

## Materials and methods

### Human EC tissue samples

A total of 58 EC tissues and 32 normal endometrial tissues were obtained from patients who were undergoing complete or partial surgical resection at the Department of Gynecology and Obstetrics, Shengjing Hospital of China Medical University during 2017–2019. No patients had received pre-operative chemotherapy, radiotherapy, and other related anti-tumor therapies. Samples were classified based on the International Federation of Obstetrics and Gynecology (FIGO 2009) staging system. All diagnoses were confirmed by two expert gynecologic pathologists. Informed consent was obtained, and the study was approved by the Ethics Committee of Shengjing Hospital of China Medical University.

### Cell culture

The Ishikawa cells were grown as an adherent monolayer in RPMI 1640 medium (Bioind, Kibbutz Beit Haemek, Israel) with 10% fetal bovine serum (Biological Industries, Kibbutz Beit Haemek, Israel), and 50 mg/mL streptomycin (Invitrogen, Carlsbad, CA).

The EC stem cells (ECSCs) were extracted from parental Ishikawa cells in serum-free medium (SFM), containing DMEM/F12 (1:1), 5% bovine serum albumin (BSA) (Roche, Basel, Switzerland), 1% Insulin-Transferrin-Selenium, 2% B27 supplements (Gibco), 20 ng/ml epidermal growth factor (EGF) and basic fibroblast growth factor (bFGF) (Peprotech, Rocky Hill, USA), and 1% penicillin-streptomycin (Gibco) and plated in a six-well low attachment surface well plate (Corning, NY, USA). All cells were maintained in a humidified incubator at 37 °C in the presence of 5% CO2.

### Flow cytometry

The cell spheres were induced and then isolated from the Ishikawa cells as described above. The single-cell suspension (100 µl of buffer per 10^7^ cells) was prepared and stained with PE-CD133 and PE-Cy7-CD44 (BD, Franklin Lakes, NJ, USA) for 30 min in the dark at 4 °C for FACS sorting on AriaIII (BD). The obtained CD44^+^/CD133^+^ cells were cultured in stem cell media as described previously.

### Transfection of cells

shRNA sequences targeting UPF1, the overexpression plasmid (GV219-UPF1, GV219-LINC00963), knock-down plasmid (GV112-LINC00963), and their respective negative control (NC) counterparts were synthesized (GeneChem, Shanghai, China). The agomir, antagomir, and their respective scrambled negative control RNAs were purchased from GenePharma (Shanghai, China). Lipofectamine 3000 (Invitrogen) was used to transfect cells with the plasmids and miRNAs for the following experiments based on the manufacturer’s instructions. The sequences of the shRNA clone, plasmids, agomir, and antagomir are listed in Supplementary Table S1.

### RNA extraction and quantitative real-time PCR

Total RNA was extracted from tissues or cells using TRIzol (Takara, Dalian, China). LncRNAs and mRNAs were reverse transcribed to the complementary DNA (cDNA) using a PrimeScript RT-polymerase (Takara, Dalian, China). The cDNAs from the miRNAs were synthesized with miRNA 1st Strand cDNA Synthesis SuperMix (Vazyme, Nanjing, China). SYBR Green Premix (Takara) on a 7500 real-time PCR system (Applied Biosystems) was used to perform quantitative real-time PCR (qRT-PCR) or miRNA Universal SYBR^®^ qPCR Master Mix (Vazyme) with specific PCR primers (Sangon Bio-tech Co., Ltd, Shanghai, China). Glyceraldehyde-3-phosphate dehydrogenase (GAPDH) and RNU6 (U6) were selected as internal references. Relative quantification (2 − ΔΔCt) method was used for fold-change calculation. The primer sequences are listed in Supplementary Table S2.

### Protein extraction and western blotting

Total protein was extracted from tissues or cells using the Protein Extraction kit (Beyotime Biotechnology, Shanghai, China). Samples were separated by 10% sodium dodecyl sulphate (SDS)-polyacrylamide gel electrophoresis (PAGE) and electrophoretically transferred to polyvinylidene difluoride (PVDF) membranes (Millipore, MA, USA). The membranes were incubated in diluted primary antibodies overnight at 4 °C (the primary antibodies are listed in Supplementary Table S3). After incubation with secondary antibodies (Goat anti-rabbit or Goat anti-mouse, 1:5000 respectively; Proteintech, Hangzhou, China), membranes were visualized with Quantity One imaging software (Bio-Rad, California, USA). ImageJ software v.1.48 was used to measure the relative integrated density values (IDVs) based on Tubulin (1:4000; Proteintech, Hangzhou, China) as an internal control.

### RNA immunoprecipitation-seq and RIP assay

RNA immunoprecipitation-sequencing (RIP-seq) was used to analyze the lncRNAs binding to UPF1 (SeqHealth Technology Company, Wuhan, China). RNA was enriched with UPF1 antibody (1:50, Abcam, Cambridge, UK) in ECSCs. The enriched and purified RNA was then broken into short fragments. Sequencing was performed on Illumina HiSeq platform after library quality tests were passed. Subsequent bioinformatics Gene Ontology (GO) and Kyoto Encyclopedia of Genes and Genomes (KEGG) analysis was conducted using DAVID bioinformatics resources (http://david.niaid.nih.gov).

RIP assays were carried out according to the instructions in the Magna RIP RNA-Binding Protein Immunoprecipitation Kit (Millipore, Bedford, MA). Whole-cell lysate of ECSCs was incubated with RIP buffer containing magnetic beads conjugated with antibody against UPF1 or negative control normal rabbit IgG for 6 h at 4 °C. Samples were treated with proteinase K and RNaseA. The immunoprecipitated RNA was examined by qRT-PCR and western blotting analyses.

### Fluorescence in situ hybridization and Immunofluorescence

Cells were fixed in paraformaldehyde (Solarbio, Beijing, China) and permeabilized in 0.5% Triton X-100. For Fluorescence in situ hybridization (FISH) assay, Cy3-labeled probes of LINC00963 were utilized to examine the expression in Ishikawa cells and ECSCs. Hybridization was performed with a Fluorescent in Situ Hybridization Kit (RIBO Bio, Guangzhou, PR China) for 12 h away from light. For Immunofluorescence (IF) assay, Ishikawa cells and ECSCs were treated with antibodies against UPF1 (1:500, Abcam, Cambridge, UK) for 2 h then labeled with Alexa 488-conjugated secondary antibody (1:400) for 1 h. The nuclei of Ishikawa cells and ECSCs were subsequently stained with 4′,6-diamidino-2-phenylindole (DAPI, Beyotime, China). The fluorescence images were captured by the confocal laser microscope (Olympus Optical, Tokyo, Japan).

### Sphere formation assay

The spheres of ECSCs were cultured in stem cell media in 6-well Ultra-Low Attachment Plates for 14 days. 0.5 ml fresh SFM was replenished every other day. The primary spheres were harvested, filtered through 40 μm cell strainers, and re-plated as above to generate 7-day-old spheres as secondary spheres. In sphere assay with xenograft-derived cell suspensions, primary and secondary spheres were counted after 4 days. For quantitation, the spheres were analyzed by sphere diameter, and the number of spheres larger than 50 μm was counted under the microscope.

### RNA stability measurement

In all, 12 h after transfection, cells were treated with the medium containing 5 μg/ml actinomycin D. Total RNA was independently collected at 0, 2, 4, 6, 8, and 10 h after actinomycin D treatment. The stability of LINC00963 was measured by qRT-PCR.

### Cell viability assay (CCK8 assay)

Cells were grown in 96-well plates. Then 10 μL of CCK-8 (Cell Counting Kit-8; Dojindo, Japan) were added into per well. After incubation at 37 °C in 5% CO2 for 3 h, the OD450 value of each well was measured with a microplate reader (Bio-Rad, Hercules, CA, USA) at 0, 24 h, 48 h, and 72 h.

### Cell migration and invasion assay

Transwell chambers (Corning, NY, USA) with a pore size of 8 µm were used to detect cell migration and invasion. Cells were resuspended in 200 μL serum-free medium and were placed into the upper chamber (or precoated with Matrigel solution (BD, Franklin Lakes, NJ, USA) for cell invasion assay). The lower chamber contained 10% FBS. After incubation for 24 h, migrated and invaded cells on the lower membrane surface were fixed with 4% paraformaldehyde and stained with 0.1% crystal violet. Three random fields were counted and cell numbers were calculated by Image J software.

### Luciferase reporter assay

The predicted binding sequence of miR-508-5p in LINC00963 (or SOX2-3′UTR) gene and its mutant sequence were amplified by PCR and cloned into a pmirGLO Dual-luciferase miRNA Target Expression Vectors (GenePharma). HEK-293T cells were seeded in 96-well plates and cotransfected with wild-type pmirGLO-LINC00963, mutant-type pmirGLO-LINC00963 (or SOX2-3′UTR-Wt, SOX2-3′UTR-Mut) reporter plasmid, and agomir-508-5p. After 48 h of cell transfection, the detection of luciferase activity was conducted using the Dual-Luciferase Reporter Assay System (Promega, Madison, WI, USA). Each group was analyzed in triplicate.

### Chemosensitivity assay

Cells were treated with 2, 4, 8, 16, 32, 64, 128 μg/mL carboplatin for 48 h, and CCK8 assays were used to detect cell growth. 36 and 66 μg/mL of carboplatin for ECCs and ECSCs, respectively, were selected based on the IC50 for subsequent chemotoxicity assays. The relative cell survivor rate was obtained by the following formula: Relative Cell Survivor Rate = (OD value of the carboplatin (+) group/OD value of the carboplatin (−) group) × 100%.

### Cell cycle analysis

Transfected cells were harvested and fixed with 70% ethanol at 4 °C overnight. The cells were mixed with 100 μL of RNase and 400 μl of propidium iodide. Then the cell cycle distribution was assessed by flow cytometry (FACScan, BD Biosciences, USA).

### Apoptosis assay

After transfection, 10^6^cells per group were washed and stained with Annexin V-APC/PI (KeyGen Biotech, Nanjing, China). After incubation for 15 min away from light, the proportion of apoptotic cells was investigated using flow cytometry (BD FACSCalibur, NJ, USA).

### Tumor xenografts in nude mice

Animal experiments were conducted in strict accordance with the protocol approved by the Scientific Research and New Technology Ethical Committee of the Shengjing Hospital of China Medical University. In all, 4- to 6-week-old female BALB/C athymic nude mice were purchased from Changsheng biotechnology (Shenyang, China). For the limiting dilution tumor formation assay, the nude mice were injected subcutaneously in the axillae with ECSCs transfected with sh-NC (5 × 10^3^, 5 × 10^2^, 100, 10, or 1) and sh-UPF1 (5 × 10^5^, 5 × 10^4^, 5 × 10^3^, 5 × 10^2^, or 100) with a 36-day monitoring period. For examining the in vivo roles of UPF1, LINC00963, and mir-508-5p, the nude mice were injected subcutaneously in the axillae with 1 × 10^6^ ECCs and 5 × 10^3^ ECSCs. Tumor volume was evaluated every 4 days and calculated according to the following formula: tumor volumes (mm^3^) = length × width^2^/2. After 28 days, mice were sacrificed. For mice survival, the number of survived nude mice was registered and survival analysis was determined with Kaplan–Meier survival curve. The dissected tumors were fixed at 4 °C with paraformaldehyde and embedded in paraffin. Tumor sections were prepared for apoptosis detection using a TUNEL Bright Green Apoptosis Detection kit (Vazyme, Nanjing, China) and for immunohistochemical analysis according to the protocols.

### Statistical analysis

Data are presented as means ± standard deviation (SEM). Statistical analyses were performed with GraphPad Prism 7.0 software (La Jolla, CA) and SPSS 23.0 software (Abbott Laboratories, Chicago, IL). Differences were analyzed with the Student’s t-test or one-way ANOVA. Values of *P* < 0.05 were defined as statistically significant.

## Results

### UPF1 is upregulated in EC tissues and functioned as a signature gene of ECSCs

**Fig. 1:**
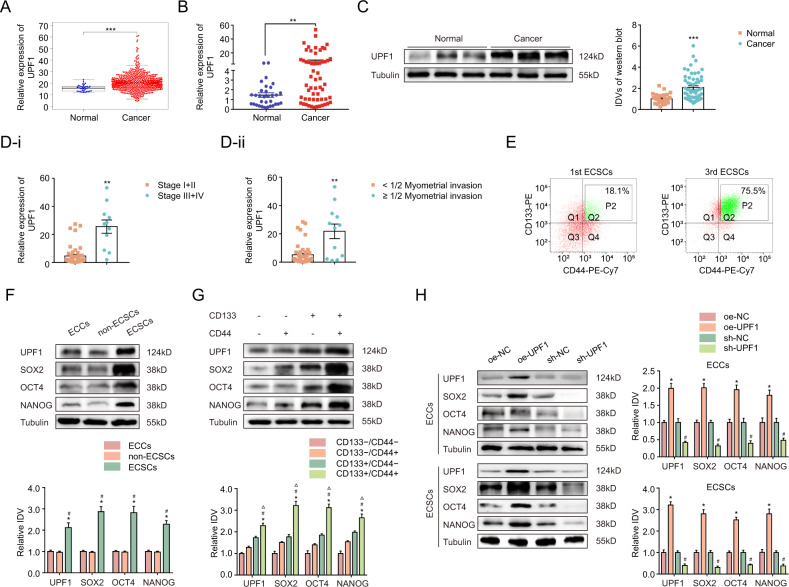
UPF1 is upregulated in EC tissues and functioned as a signature gene of ECSCs. **A** UPF1 expression levels in EC tissues (*N* = 546) and normal tissues (*n* = 33) in the TCGA cohort. **B** UPF1 expression levels in EC tissues (*n* = 58) compared to normal tissues (*n* = 32) detected using qRT-PCR. Data are presented as the means ± SEM, **P < 0.01. **C** UPF1 expression levels in EC tissues (*n* = 58) compared to normal tissues (*n* = 32) detected using western blotting. Data are presented as the means ± SEM, ***P < 0.001. **D** UPF1 expression levels in patients with different (**D**-i) tumor stages and (**D**-ii) myometrial invasion in EC tissues (*n* = 58) compared to normal tissues (*n* = 32). Data are presented as the means ± SEM, **P < 0.01. **E** Flow cytometry analysis of CD133^+^/CD44^+^ cells sorted from the 1st and 3rd ECSCs. **F** The expression of UPF1, SOX2, OCT4, and NANOG in ECCs, non-ECSCs, and ECSCs detected using western blotting. The results are presented as the ratio of the integrated density values of UPF1, SOX2, OCT4, and NANOG versus Tubulin. The graphs represent the alteration in relation to ECCs (protein of interest/Tubulin equal to 1). Data are presented as the means ± SEM (*n* = 3, each group), *P < 0.05 vs. ECCs group, #*P* < 0.05 vs. non-ECSCs group. **G** The expression of UPF1, SOX2, OCT4, and NANOG in CD133^−^/CD44^−^, CD133^−^/CD44^+^, CD133^+^/CD44^−^, and CD133^+^/CD44^+^ cells detected using western blotting. The results are presented as the ratio of the integrated density values of UPF1, SOX2, OCT4, and NANOG versus Tubulin and the graphs represent the alteration in relation to the CD44−/CD133− cells (protein of interest/Tubulin equal to 1). Data are presented as the means ± SEM (*n* = 3, each group), *P < 0.05 vs. CD133^−^/CD44^−^ group, #*P* < 0.05 vs. CD133^−^/CD44^+^ group, △*P* < 0.05 vs. CD133^+/^CD44^−^ group. **H** Effect of UPF1 overexpression or knockdown on SOX2, OCT4, and NANOG expression assessed using western blotting. The results are presented as the ratio of the integrated density values of UPF1, SOX2, OCT4, and NANOG versus Tubulin. The graphs represent the alteration in the oe-UPF1 group and the sh-UPF1 group relative to their respective control groups (protein of interest/Tubulin equal to 1). Data are presented as the means ± SEM, *P < 0.05 vs. oe-NC group, #*P* < 0.05 vs. sh-NC group.

Analysis of the TCGA dataset (https://tcga-data.nci.nih.gov/tcga/) revealed a high expression of UPF1 in EC tissues (*N* = 546) compared to normal endometrial tissues (*n* = 33) (Fig. 1A). To confirm this finding, we examined 58 EC samples and 32 unaffected tissues using qRT-PCR and western blotting and observed that UPF1 expression was elevated in EC specimens (Fig. 1B, C). UPF1 expression was significantly associated with tumor stage, race, age, weight, menopause, histological subtype, and TP53 mutation status from the TCGA cohort (Supplementary Fig. S1A). Based on specimens collected in our hospital, we found that high UPF1 expression correlated with advanced clinical stage and myometrial invasion >50% (Fig. 1D). UPF1 was expressed in various EC cell lines (Supplementary Fig. S1B). Ishikawa, which has been previously described as the most appropriate cell to induce ECSCs of the commonly used EC cell lines, was chosen as the representative in our experiments [32]. To determine whether UPF1 was related to ECSCs, CD133^+^/CD44^+^ cells, which exhibit CSC features, were cultured in suspension from parental Ishikawa cells followed by flow cytometry sorting. The sorting efficiency was 18.1% ± 0.5% for first-generation ECSCs and 75.5% ± 1.1% for third-generation ECSCs (Fig. 1E and Supplementary Fig. S2). It is well-accepted that 3rd ECSCs possess enhanced stemness properties considering higher self-renewal and tumorigenicity in vivo compared to 1st ECSCs [9]. Thus, we selected 3rd ECSCs for subsequent studies. Our data revealed that UPF1 expression was significantly elevated in ECSCs compared to parental cells or non-ECSCs (CD133^−^/CD44^−^ cells) (Fig. 1F). Moreover, UPF1 showed the strongest expression in the CD133^+^/CD44^+^ subpopulation of the sorted four subsets (Fig. 1G). The expression of stemness-associated transcription factors (SOX2, OCT4, and NANOG) was upregulated in UPF1-overexpressing cells and downregulated by UPF1 silencing (Fig. 1H). Together, these findings suggested UPF1 as a candidate biomarker for ECSCs.

### UPF1 is required to maintain the ECSC phenotype

**Fig. 2:**
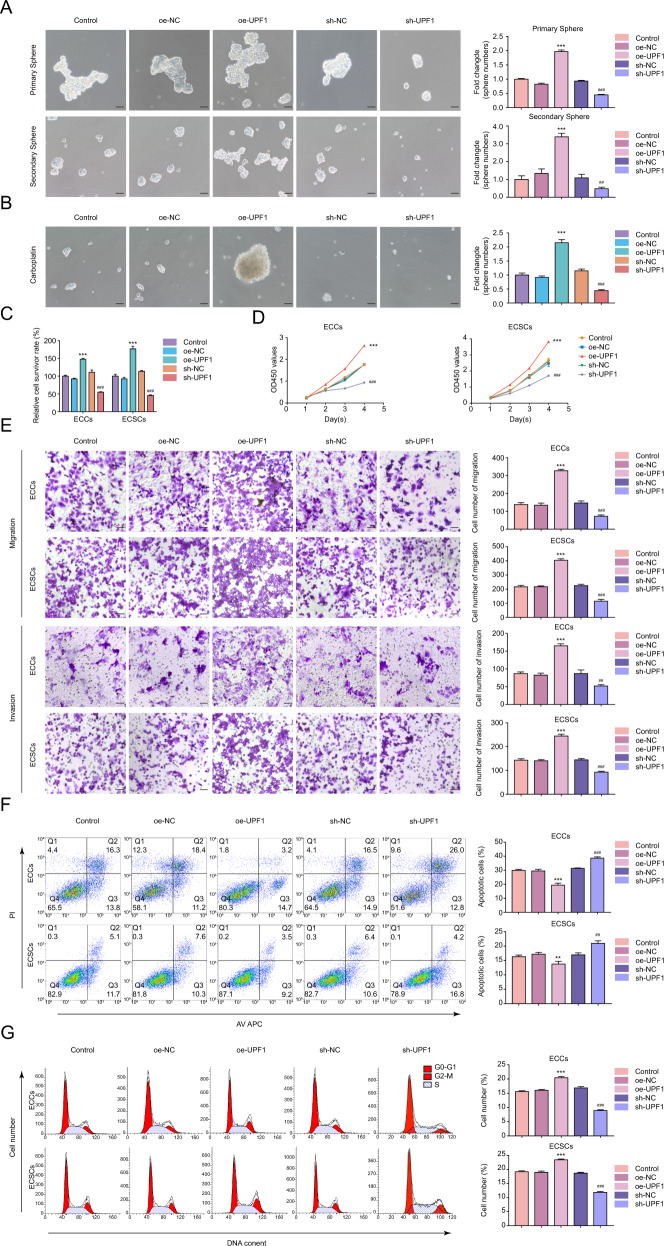
UPF1 is required to maintain the ECSC phenotype. **A** Effects of UPF1 on self-renewal capacity assessed using serial sphere formation assay. **B** Effects of UPF1 on carboplatin resistance assessed using the sphere formation assay. **C** Effects of UPF1 on carboplatin resistance assessed by the CCK8 assay. **D** Effects of UPF1 on proliferation assessed by the CCK8 assay. **E** Effects of UPF1 on migration and invasion assessed using the Transwell assay. **F** Effects of UPF1 on apoptosis assessed using flow cytometry analysis. **G** Effects of UPF1 on cell cycle progression assessed using flow cytometry analysis. Data are presented as the means ± SEM (*n* = 3, each group), **P < 0.01, ***P < 0.001 vs. oe-NC group, ##*P* < 0.01, ###*P* < 0.001 vs. sh-NC group. Scale bars, 50 μm.

The biological behaviors were detected to further examine the function of UPF1 in ECSCs and ECCs (transfection efficiency of UPF1 is shown in Supplementary Fig. S3A–D). Sh-UPF1-1 and sh-UPF1-2 presented better knockdown efficiency and were selected for subsequent loss-of-function assays (Supplementary Figs. S3C, D and S4). A defining property of CSCs is self-renewal. Therefore, the sphere formation assay was used to address this issue. As shown in Fig. 2A, UPF1 overexpression greatly increased the clonogenic potential of ECSCs in the primary and secondary spheres, and UPF1 deficiency reduced the clonogenic potential. Chemotherapy resistance is another hallmark of CSCs. Since the behavior of ECSCs in carboplatin has never been investigated, we selected carboplatin as a representative chemotherapeutic agent. Our data showed that the IC50 value of ECCs (36.5 μg/mL) was less than ECSCs (66.6 μg/mL) (Supplementary Fig. S5). UPF1 overexpression mitigated the toxicity of carboplatin in the sphere assay and CCK-8 assay, and UPF1 knockdown enhanced the cytotoxic effects (Fig. 2B, C). The role of UPF1 in proliferation was investigated. We confirmed that the proliferation of cells was increased in the oe-UPF1 group and decreased in the sh-UPF1 group (Fig. 2D). The migration and invasion abilities of ECSCs were examined using the Transwell assay, and the ability was higher in the oe-UPF1 group than the oe-NC group. In contrast, UPF1 knockdown decreased this ability (Fig. 2E). We analyzed the cell cycle regulation and apoptosis of UPF1. UPF1 overexpression decreased the proportion of apoptotic cells relative to the control, and the proportion was increased in the sh-UPF1 group (Fig. 2F). UPF1 overexpression improved the fraction of ECSCs in G2–M phase, and UPF1 knockdown decreased the cell number (Fig. 2G). Similar but less pronounced results were observed in ECCs. Briefly, UPF1-mediated tumorigenicity in EC was primarily attributed to the self-renewal capacity of ECSCs, and silencing of UPF1 exerted tumor-suppressive effects on ECSCs.

### UPF1 binds and positively regulates LINC00963

**Fig. 3:**
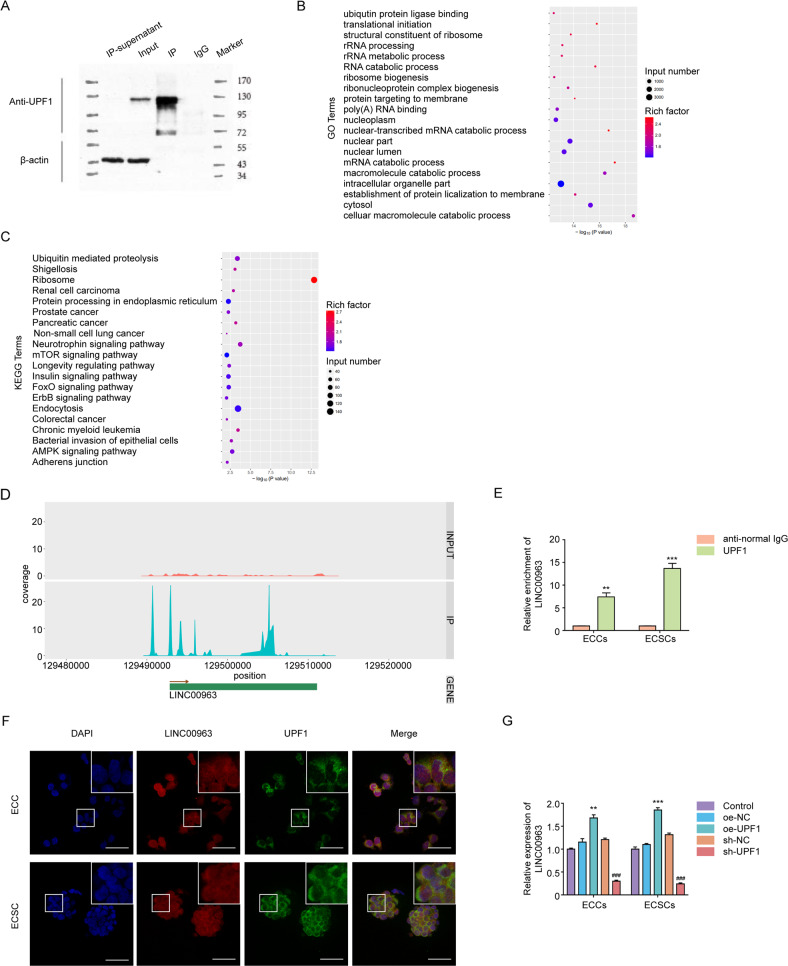
UPF1 binds and positively regulates LINC00963. **A** Western blotting of UPF1 immunoprecipitation. **B** Top 20 upregulated GO and **C** Top 20 upregulated KEGG metabolic pathways of peak-associated genes. **D** Binding sites and enrichment of UPF1 and LINC00963. **E** Binding of UPF1 and LINC00963 determined using the RIP assay. **F** Co-localization of UPF1 and LINC00963 determined using RNA-FISH and IF. Data are presented as the means ± SEM, ***P < 0.001. **G** Effect of UPF1 overexpression or knockdown on LINC00963 expression assessed using qRT-PCR. Data are presented as the means ± SEM (*n* = 3, each group), **P < 0.01, ***P < 0.001 vs. oe-NC group, ###*P* < 0.001 vs. sh-NC group. Scale bars, 50 μm.

To delve further into the potential mechanism of the UPF1 effect on ECSC activity, RIP‑seq was used to analyze lncRNAs binding to UPF1 in ECSCs. The IP and input lanes showed bands for UPF1 antibody, which indicated that IP was successful (Fig. 3A). A total of 332 lncRNA loci were identified (Supplementary Table S4). GO and KEGG analyses demonstrated that these lncRNAs were involved in numerous biological processes and signaling pathways, such as cancer metabolism (Fig. 3B, C). The top 7 lncRNAs with relatively higher enrichment are shown in Supplementary Fig. S6A, and LINC00963 exhibited the greatest content difference between ECCs and ECSCs and was selected for further exploration (Supplementary Fig. S6B and Fig. 3D). The binding fragments and intensities of UPF1 and LINC00963 are also shown in Supplementary Table S5. RIP was performed to further validate the potential direct binding of LINC00963 to UPF1 in ECSCs (Fig. 3E). The colocalization of UPF1 protein and LINC00963 in the cytoplasm further confirmed their interaction (Fig. 3F). We observed an increase in LINC00963 following UPF1 overexpression and the opposite results were observed with UPF1 depletion using qRT-PCR (Fig. 3G). These results collectively indicated that LINC00963 was involved in UPF1-mediated regulation of ECSCs.

### LIN00963 enhances the tumorigenicity of ECSCs

**Fig. 4:**
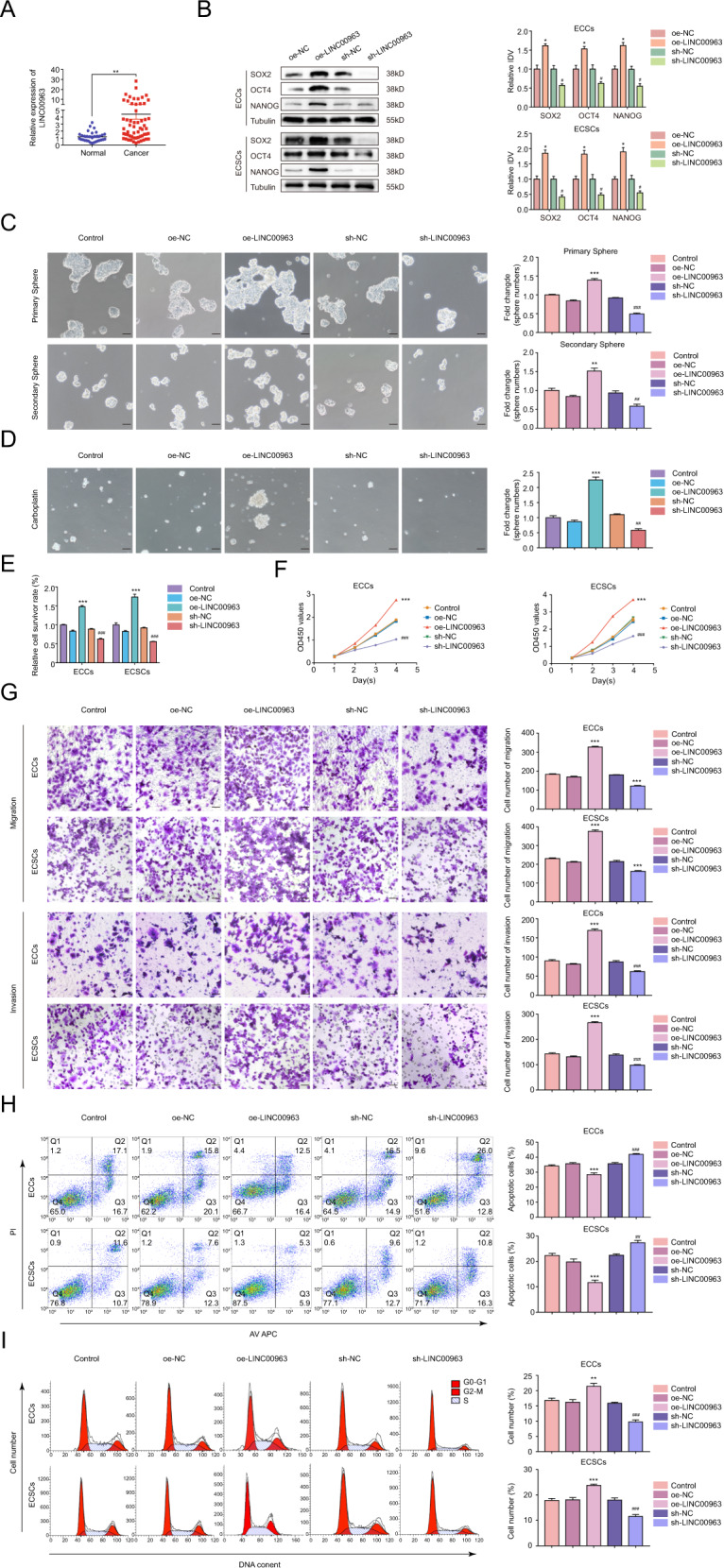
LIN00963 enhances the tumorigenicity of ECSCs. **A** LIN00963 expression levels in EC tissues (*n* = 58) compared to normal tissues (*n* = 32) detected using qRT-PCR. Data are presented as the means ± SEM, **P < 0.01. **B** Effects of LIN00963 overexpression or knockdown on SOX2, OCT4, and NANOG expression assessed using western blotting. The results are presented as the ratio of the integrated density values of SOX2, OCT4, and NANOG versus Tubulin. The graphs represent the alteration in the oe-LIN00963 group and the sh-LIN00963 group relative to their respective control groups (protein of interest/Tubulin equal to 1). **C** Effects of LIN00963 on self-renewal capacity assessed using serial sphere formation assay. **D** Effects of LIN00963 on carboplatin resistance assessed using the sphere formation assay. **E** Effects of LIN00963 on carboplatin resistance assessed using the CCK-8 assay. **F** Effects of LIN00963 on proliferation assessed using the CCK-8 assay. **G** Effects of LIN00963 on migration and invasion assessed using the Transwell assay. **H** Effects of LIN00963 on apoptosis assessed using flow cytometry analysis. **I** Effects of LIN00963 on cell cycle progression assessed using flow cytometry analysis. Data are presented as the means ± SEM (*n* = 3, each group), *P < 0.05, **P < 0.01, ***P < 0.001 vs. oe-NC group, #*P* < 0.05, ##*P* < 0.01, ###*P* < 0.001 vs. sh-NC group. Scale bars, 50 μm.

The expression of LINC00963 in EC and normal tissues were detected using qRT-PCR. As shown in Fig. 4A, LINC00963 was significantly upregulated in EC tissues and positively correlated with clinical stage, myometrial invasion, and lymph node metastasis (Supplementary Table S6). The transfection efficiency of LINC00963 is shown in Supplementary Fig. S7. Notably, LINC00963 overexpression resulted in the upregulation of SOX2, OCT4, and NANOG, and LINC00963 knockdown reduced the expression of SOX2, OCT4, and NANOG (Fig. 4B). LINC00963 overexpression dramatically augmented primary and secondary sphere formation of ECSCs (Fig. 4C), induced carboplatin resistance (Fig. 4D, E), boosted cell proliferation (Fig. 4F), enhanced cell migration and invasion (Fig. 4G), inhibited apoptosis (Fig. 4H), and promoted cells to enter the G2-M phase of the cell cycle (Fig. 4I). LINC00963 knockdown yielded opposing effects, and these effects were stronger than in the parental ECCs. Taken together, these data demonstrated that overexpressed LINC00963 induced a stem cell-like state in the parental cells and promoted the stemness in ECSCs.

### UPF1 regulates the biological behavior of ECSCs by stabilizing LINC00963

**Fig. 5:**
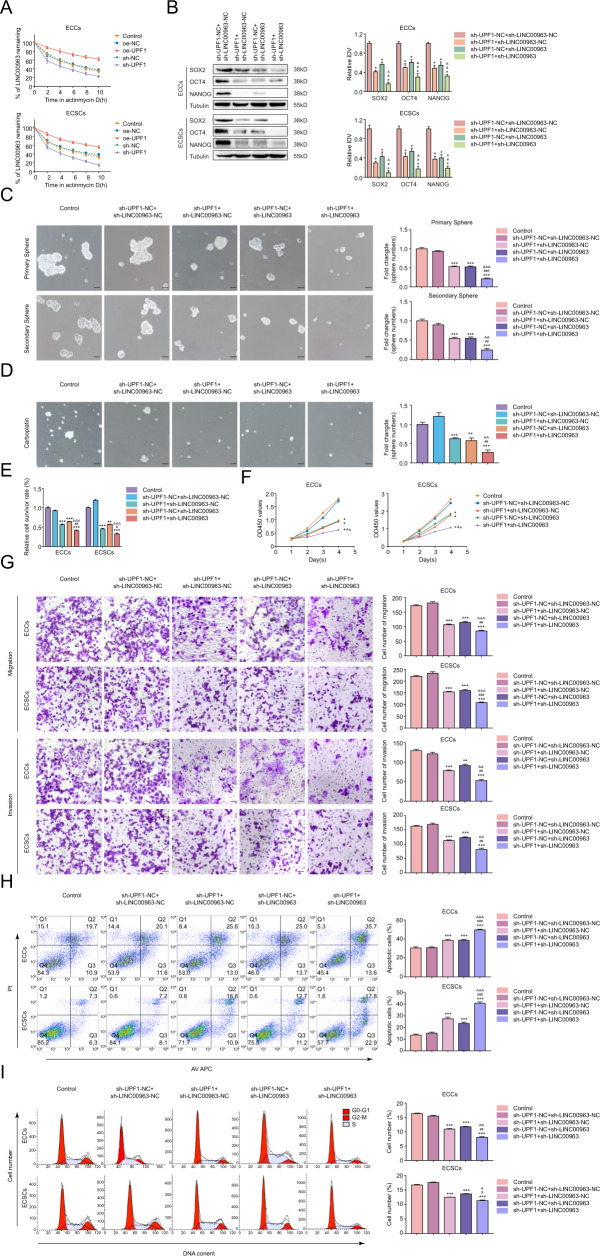
UPF1 regulates the biological behavior of ECSCs by stabilizing LINC00963. **A** LIN00963 RNA half-life measured using qRT-PCR after actinomycin D treatment. **B** Effects of UPF1 and LIN00963 inhibition on SOX2, OCT4, and NANOG expression assessed using western blotting. The results are presented as the ratio of the integrated density values of SOX2, OCT4, and NANOG versus Tubulin. The graphs represent the alteration in relation to the sh-UPF1-NC + sh-LINC00963-NC group (protein of interest/Tubulin equal to 1). **C** Effects of UPF1 and LIN00963 inhibition on self-renewal capacity assessed using serial sphere formation assay. **D** Effects of UPF1 and LIN00963 inhibition on carboplatin resistance assessed using the sphere formation assay. **E** Effects of UPF1 and LIN00963 inhibition on carboplatin resistance assessed using the CCK8 assay. **F** Effects of UPF1 and LIN00963 inhibition on proliferation assessed using the CCK8 assay. **G** Effects of UPF1 and LIN00963 inhibition on migration and invasion assessed using the Transwell assay. **H** Effects of UPF1 and LIN00963 inhibition on apoptosis assessed using flow cytometry analysis. **I** Effects of UPF1 and LIN00963 inhibition on cell cycle progression assessed using flow cytometry analysis. Data are presented as the means ± SEM (*n* = 3, each group), *P < 0.05, **P < 0.01, ***P < 0.001 vs. sh-UPF1-NC + sh-LINC00963-NC group, #*P* < 0.05, ##*P* < 0.01, ###*P* < 0.001 vs. sh-UPF1 + sh-LINC00963-NC group, △*P* < 0.05, △△*P* < 0.01, △△△*P* < 0.001 vs. sh-UPF1-NC + sh-LINC00963 group. Scale bars, 50 μm.

We subsequently investigated the mechanism of UPF1 regulation of LINC00963. We observed a markedly prolonged half-life in oe-UPF1 cells and a shortened half-life in sh-UPF1 cells, which indicated that UPF1 delayed the degradation of LINC00963 (Fig. 5A). Compared to the sh-UPF1-NC + sh-LINC00963-NC group, inhibition of UPF1, inhibition of LINC00963, or inhibition of UPF1 combined with inhibition of LINC00963 significantly reduced the expression of SOX2, OCT4, and NANOG (Fig. 5B), impaired tumor sphere formation (Fig. 5C), increased the cytotoxic effect of carboplatin (Fig. 5D, E), attenuated cell proliferation (Fig. 5F), impeded cell migration and invasion (Fig. 5G), promoted apoptosis (Fig. 5H), and decreased the cell numbers in G2–M phase of the cell cycle (Fig. 5I). Moreover, ECSCs treated with sh-UPF1 and sh-LINC00963 exhibited poorer self-renewal, resistance to carboplatin, proliferation, migration and invasion capability, a higher apoptosis ratio, and even more severe cell cycle arrest. These data suggested that UPF1 facilitated malignant progression of ECSCs by stabilizing LINC00963.

### MiR-508-5p is a target of LINC00963 and functions as a tumor suppressor in ECSCs

**Fig. 6:**
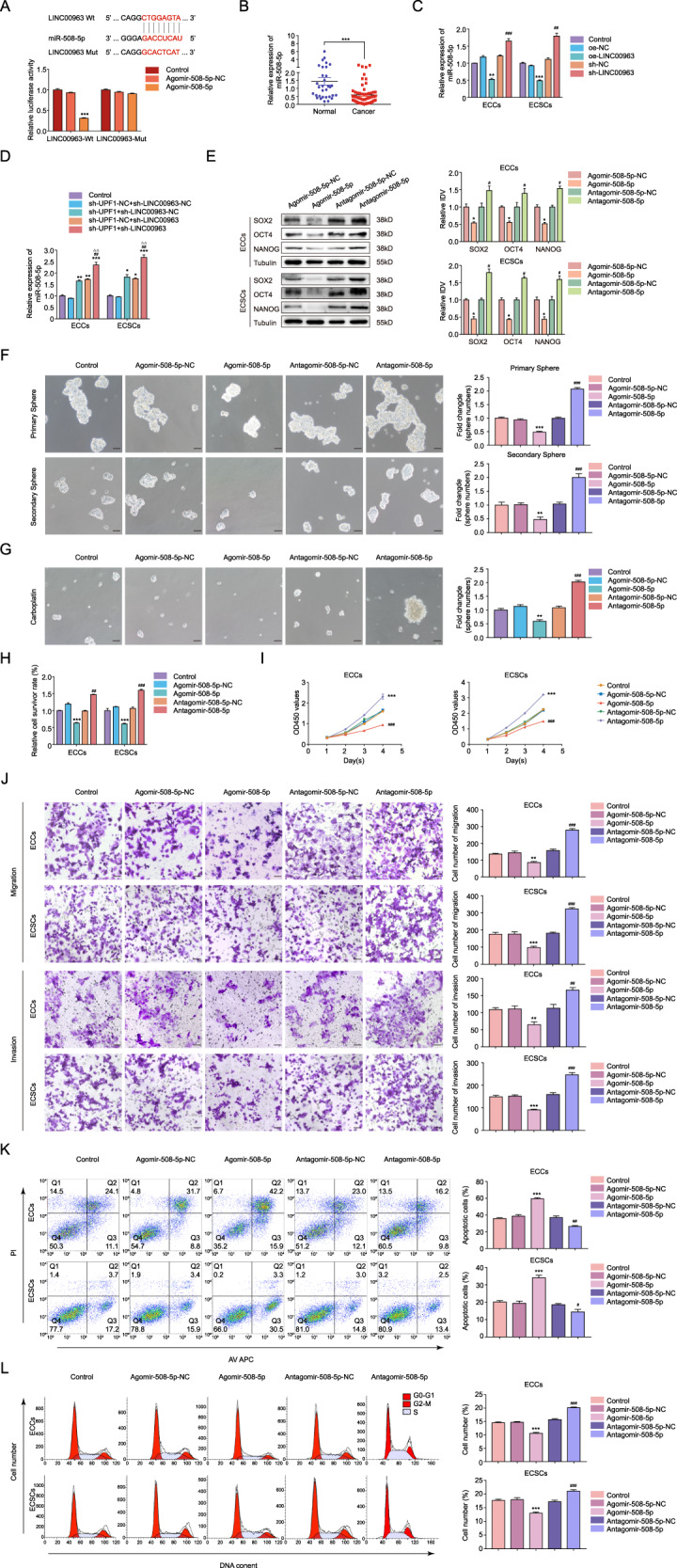
MiR-508-5p is a target of LINC00963 and functions as a tumor suppressor in ECSCs. **A** The predicted miR-508-5p binding sites in the 3′UTR region of LINC00963 (LINC00963-Wt) and the designed mutant sequence (LINC00963-Mut) are indicated. Relative luciferase activity was conducted after cells were transfected with LINC00963-Wt or LINC00963-Mut, ***P < 0.001 vs. LINC00963-Wt+Agomir-508-5p-NC group. **B** MiR-508-5p expression levels in EC tissues (*n* = 58) compared to normal tissues (*n* = 32) detected using qRT-PCR, ***P < 0.001. **C** Effects of LIN00963 overexpression or knockdown on miR-508-5p expression assessed using qRT-PCR, **P < 0.01, ***P < 0.001 vs. oe-NC group, ##*P* < 0.01, ###*P* < 0.001 vs. sh-NC group. **D** Effects of UPF1 and LIN00963 inhibition on miR-508-5p expression assessed using qRT-PCR, *P < 0.05, **P < 0.01, ***P < 0.001 vs. sh-UPF1-NC + sh-LINC00963-NC group, ##*P* < 0.01 vs. sh-UPF1 + sh-LINC00963-NC group, △△*P* < 0.01 vs. sh-UPF1-NC + sh-LINC00963 group. **E** Effect of miR-508-5p overexpression or knockdown on SOX2, OCT4, and NANOG expression assessed using western blotting. The results are presented as the ratio of the integrated density values of SOX2, OCT4, and NANOG versus Tubulin. The graphs represent the alteration in the agomir-508-5p group and the antagomir-508-5p group relative to their respective control groups (protein of interest/Tubulin equal to 1). **F** Effects of miR-508-5p on self-renewal capacity assessed using serial sphere formation assay. **G** Effects of miR-508-5p on carboplatin resistance assessed using the sphere formation assay. **H** Effects of miR-508-5p on carboplatin resistance assessed using the CCK8 assay. **I** Effect of miR-508-5p on proliferation assessed using the CCK8 assay. **J** Effect of miR-508-5p on migration and invasion assessed using the Transwell assay. **K** Effects of miR-508-5p on apoptosis assessed using flow cytometry analysis. **L** Effects of miR-508-5p on cell cycle progression assessed using flow cytometry analysis. Data are presented as the means ± SEM (*n* = 3, each group), *P < 0.05, **P < 0.01, ***P < 0.001 vs. Agomir-508-5p-NC group, #*P* < 0.05, ##*P* < 0.01, ###*P* < 0.001 vs. Antagomir-508-5p-NC group. Scale bars, 50 μm.

To further determine the detailed mechanisms of UPF1 stabilization of LINC00963 in ECSCs, the interaction between LINC00963 and downstream miRNAs was studied. Bioinformatics databases (StarBase v3.0) predicted that LINC00963 harbored a miR-508-5p binding site, and a dual-luciferase reporter assay was performed to verify this hypothesis. The luciferase activity was dramatically decreased in the LINC00963-Wt + Agomir-508-5p group compared to the Agomir-508-5p-NC group, but the luciferase activity in the LINC00963-Mut group was not affected (Fig. 6A). We analyzed the expression of miR-508-5p in EC tissues using qRT-PCR. As shown in Fig. 6B, miR-508-5p expression was drastically downregulated in EC tissues and showed negative correlations with clinical stage, myometrial invasion, and lymph node metastasis (Supplementary Table S7). We determined that inhibition of LINC00963 increased the content of miR-508-5p (Fig. 6C). Moreover, we found a significantly higher relative expression of miR-508-5p in the sh-UPF1 + sh-LINC00963 group (Fig. 6D). The qRT-PCR analysis showed that miR-508-5p overexpression led to a marked decrease in LINC00963 expression (Supplementary Fig. S8A-C). The levels of SOX2, OCT4, and NANOG, self-renewal, drug resistance, proliferation, migration, invasion, and the proportion of ECSCs in G2–M phase significantly declined, apoptosis was significantly increased in the agomir-508-5p group, and the antagomir-508-5p group exhibited the opposite effect (Fig. 6E–L). In summary, these results indicated a reciprocal suppression between LINC00963 and miR-508-5p, and miR-508-5p played a tumor suppressor role in ECSCs.

### MiR-508-5p/SOX2 axis is responsible for the stemness-promoting effects of LINC00963

**Fig. 7:**
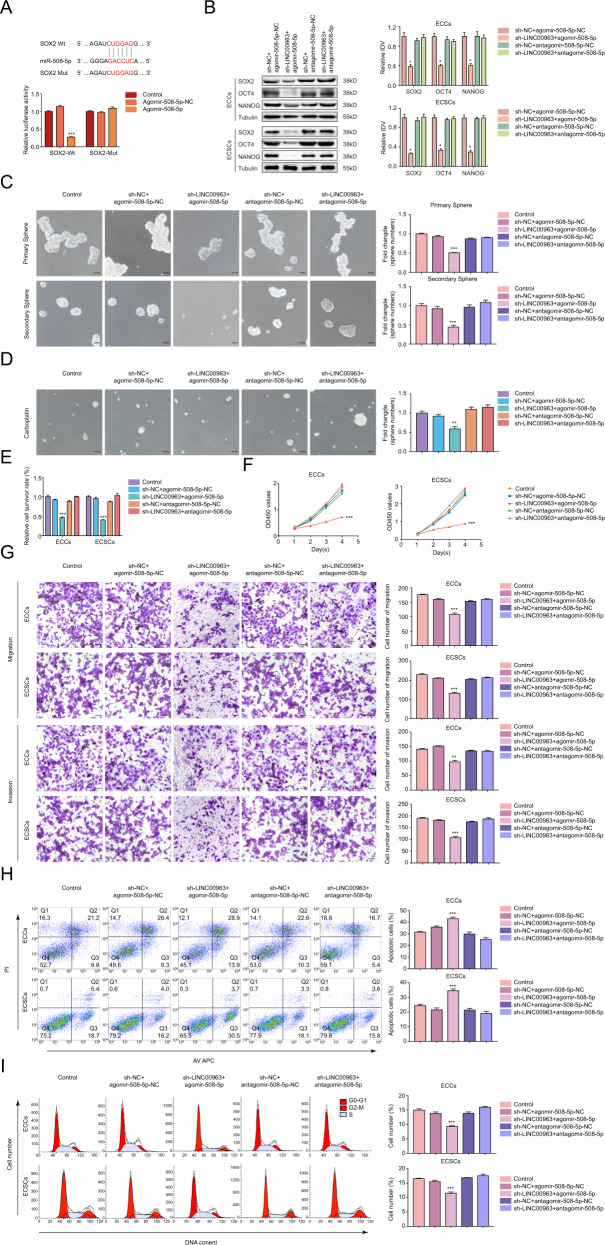
MiR-508-5p/SOX2 axis is responsible for the stemness-promoting effects of LINC00963. **A** The predicted miR-508-5p binding sites in the 3′UTR region of SOX2 (SOX2-Wt) and the designed mutant sequence (SOX2-Mut) are indicated. Relative luciferase activity was conducted after cells were transfected with SOX2-Wt or SOX2-Mut, ***P < 0.001 vs. SOX2-Wt+Agomir-508-5p-NC group. **B** Effects of LIN00963 and miR-508-5p on SOX2, OCT4, and NANOG expression assessed using western blotting. The results are presented as the ratio of the integrated density values of SOX2, OCT4, and NANOG versus Tubulin. The graphs represent the alteration in relation to the sh-NC + agomir-508-5p-NC group (protein of interest/Tubulin equal to 1). **C** Effects of LIN00963 and miR-508-5p on self-renewal capacity assessed using serial sphere formation assay. **D** Effects of LIN00963 and miR-508-5p on carboplatin resistance assessed using the sphere formation assay. **E** Effects of LIN00963 and miR-508-5p on carboplatin resistance assessed using the CCK8 assay. **F** Effects of LIN00963 and miR-508-5p on proliferation assessed using the CCK8 assay. **G** Effects of LIN00963 and miR-508-5p on migration and invasion assessed using the Transwell assay. **H** Effects of LIN00963 and miR-508-5p on apoptosis assessed using flow cytometry analysis. **I** Effects of LIN00963 and miR-508-5p on cell cycle progression assessed using flow cytometry analysis. Data were presented as the means ± SEM (*n* = 3, each group), *P < 0.05, **P < 0.01, ***P < 0.001 vs. sh-NC + agomir-508-5p-NC group.

We next examined whether other downstream mRNA targets of miR-508-5p were involved in the modulation of ECSCs. StarBase v3.0 was used to predict the binding site of miR-508-5p in the 3′UTR of SOX2, and a luciferase reporter assay was performed to clarify the interaction (Fig. 7A). Thereafter, miR-508-5p overexpression and knockdown were transfected into sh-LINC00963 cells. Compared to the sh-NC + agomir-508-5p-NC group, the expression of SOX2, sphere-forming ability, carboplatin resistance, cell proliferation, migration and invasion, and cell cycle progression prior to G2-M phase in the sh-LINC00963 + agomiR-508-5p group were markedly impaired, and the apoptosis proportion increased significantly (Fig. 7B–I). However, the biological behaviors in the sh-LINC00963 + antagomir-508-5p group did not considerably differ from the control group, which verified that miR-508-5p functionally reversed the tumor-promoting effect of LINC00963. Our data strongly suggest that LINC00963 acts as a sponge of miR-508-5p to upregulate the expression of its target, SOX2, which promotes ECSC formation and a malignant phenotype.

### UPF1 regulates ECSC initiation via the LINC00963/miR-508-5p axis in vivo

**Fig. 8:**
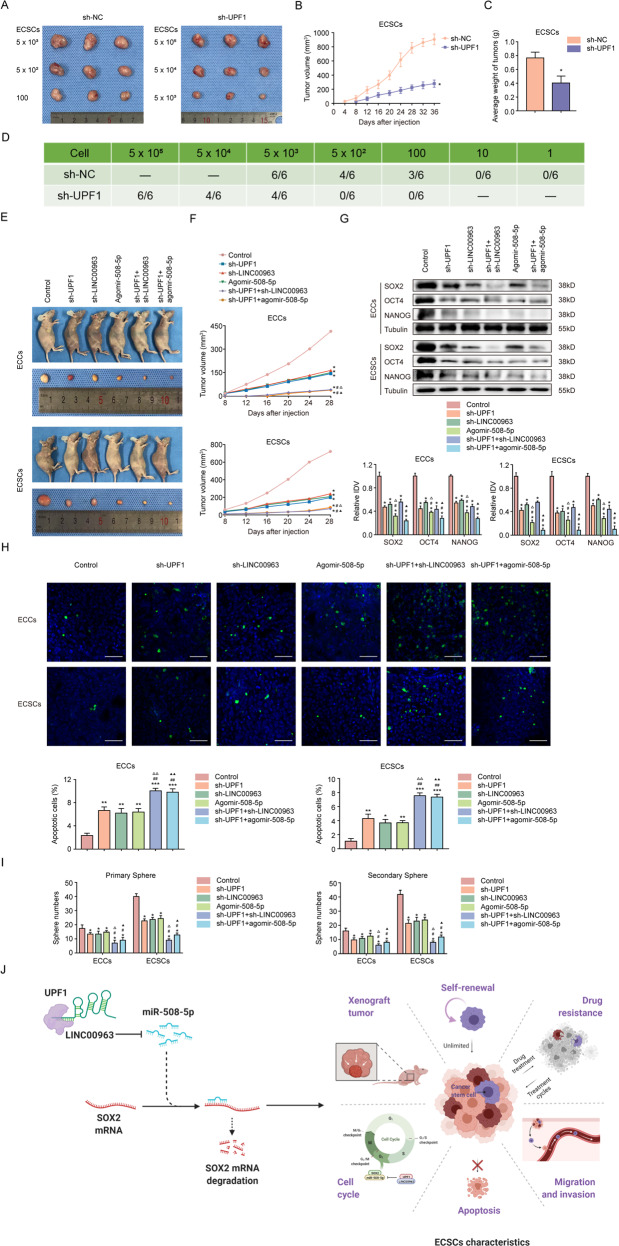
UPF1 regulates ECSC initiation via the LINC00963/miR-508-5p axis in vivo. Tumorigenicity assessed by the **A** morphology, **B** volumes, and **C** weights of xenografts formed from ECSCs transfected with sh-NC and sh-UPF1 (*n* = 3, each group). **D** Tumorigenicity initiating capacity of the minimum number of ECSCs transfected with sh-NC and sh-UPF1 (*n* = 6, each group). **E** The nude mice carrying tumors from the respective groups are shown. The sample tumors from the respective groups are shown (*n* = 3, each group). **F** Tumor growth curves are shown (*n* = 3, each group). **G** Expression of SOX2, OCT4, and NANOG from the respective groups assessed using western blotting (*n* = 3, each group). The results are presented as the ratio of the integrated density values of SOX2, OCT4, and NANOG versus Tubulin. The graphs represent the alteration in relation to the control group (protein of interest/Tubulin equal to 1). **H** Apoptosis of tumors from the respective groups detected using TUNEL assays (*n* = 3, each group). **I** The primary and secondary spheres numbers of xenograft-derived cells detected by serial sphere formation assay (*n* = 3, each group). **J** The proposed mechanism underlying the UPF1/LINC00963/miR-508-5p/SOX2 axis in ECSCs. Data are presented as the means ± SEM (*n* = 3, each group), *P < 0.05, **P < 0.01, ***P < 0.001 vs. Control group, #*P* < 0.05, ##*P* < 0.01 vs. sh-UPF1 group, △△*P* < 0.01 vs. sh-LINC00963 group, ▲*P* < 0.05, ▲▲*P* < 0.01 vs. Agomir-508-5p group. Scale bars, 50 μm.

The limiting dilution assay implied that UPF1 knockdown hindered the tumor initiation capacity of ECSCs. Specifically, 5 × 10^3^ ECSCs transfected with sh-UPF1 were required to form a xenograft tumor, while only 100 ECSCs transfected with sh-NC were enough (Fig. 8A). Tumor volume, weight, tumor growth rate, and tumor take rate (TTR) were lower for the sh-UPF1 group than the sh-NC group (Fig. 8B–D). We next determined the functions of UPF1, LINC00963, and miR-508-5p in xenograft models. Overall, the volumes of xenograft tumors formed by ECSCs were greater than the tumors formed by ECCs (Fig. 8E, F). The sh-UPF1 + sh-LINC00963 group produced the smallest tumors. The expression of SOX2, OCT4, and NANOG was the lowest, and apoptosis was highest in the sh-UPF1 + sh-LINC00963 group (Fig. 8G, H and Supplementary Fig. S9). The nude mice in the sh-UPF1 + sh-LINC00963 group exhibited the longest survival period (Supplementary Fig. S10). For further characterization, tumors were harvested, digested into cell suspensions, and subjected to sphere forming assays (Fig. 8I). The primary spheres of cells originating from ECSC xenografts were appreciably more than those from xenografts formed by ECCs, and the sh-UPF1 + sh-LINC00963 group showed the least sphere numbers. Similar results were observed in the secondary spheres. These in vivo data support the concept that UPF1 regulates ECSC initiation through the LINC00963/miR-508-5p axis.

## Discussion

CSCs are considered a culprit of cancer growth and relapse due to distinct characteristics, including self-renewal and drug resistance [7–9, 32–35], and eradication of ECSCs is the premise for a complete cure of EC. Therefore, it is critical to elucidate the mechanisms contributing to CSC maintenance. The present study focused on the functions of the RNA-binding protein UPF1 in EC and identified UPF1 as a novel driver of ECSC formation. UPF1 was highly expressed in EC tissues and ECSCs and positively related to self-renewal, carboplatin resistance, proliferation, migration, invasion, antiapoptosis, and cell cycle progression of ECSCs. Further, we found that inhibition of UPF1 suppressed the malignant phenotype of ECSCs by destabilizing the oncogene LINC00963, which acted as an endogenous competition RNA (ceRNA) to prevent the tumor suppressor miR-508-5p from binding to the 3′UTR of SOX2 mRNA. Notably, knockdown of UPF1 and LINC00963 in combination severely impaired the in vivo tumorigenic potential of ECSCs. Our study is the first study to demonstrate that UPF1 played a critical role in priming the self-renewal and tumorigenicity of ECSCs via modulation of the LINC00963/miR-508-5p/SOX2 axis.

UPF1 is best known for its central role in NMD, which stimulates the rapid destruction of aberrant RNAs harboring premature stop codons and serves as a quality control pathway [36–38]. The functions of UPF1 in tumorigenesis are intricate. UPF1 is highly expressed in a variety of cancers, such as glioblastomas [16], and low expression levels of UPF1 were documented in other types of tumors, such as gastric cancer [11], which suggest that UPF1 has pro- and antitumor effects. There are few studies on UPF1 in EC [39, 40]. One previous report showed downregulation of UPF1 in EC [40]. High expression of UPF1 was observed in EC tissues in our study, and it was related to muscular invasion and advanced FIGO stage. The cause of this discrepancy was likely due to differences in specimen source. Therefore, we chose to comprehensively explore the detailed mechanisms of UPF1 in the regulation of EC. Our data robustly demonstrated that UPF1 knockdown hindered the malignant behaviors of ECCs and inhibited tumor growth in vivo. In addition to its involvement in cancer, UPF1 also functions in several other ways. For example, UPF1 plays a direct role in DNA synthesis and genome stability in human cells [36, 37]. UPF1-null cells initiate but were unable to complete the cell cycle, which is in line with our results. The loss of UPF1 also resulted in embryonic lethality in zebrafish, flies, and mice, which indicates an essential role of UPF1 in mammalian early embryonic development [41]. Previous studies also revealed that UPF1 promoted the neural stem cell state and the maintenance of stemness in colorectal CSCs [12, 14], which is consistent with our finding that UPF1 played a pivotal role in the maintenance of the ECSC phenotype and that UPF1 knockdown suppressed ECSC initiation in vivo.

As an RBP, UPF1 participates in virtually all kinds of posttranscriptional regulatory events by establishing highly dynamic interactions with noncoding RNAs, dictating the fate of each transcript, and supporting cellular homeostasis [42]. UPF1 bound to Linc-00313 in glioma and enhanced its stability to promote proliferation, invasion, and migration [16]. In contrast, UPF1 inhibited the expression of MALAT1 in gastric cancer by accelerating its degradation [11]. Perturbations in RBP–RNA networks were also causally linked with pluripotency and the differentiation of stem cells. RBPs PTBP1, hnRNP-K, and NCL formed an RNA-multiprotein complex with the lncRNA TUNA, which was specifically enriched at the SOX2, NANOG, and FGF4 promoters to control the embryonic stem cell (ESC) state in mice [43, 44].

Because UPF1 primarily localizes to the cytoplasm, we conjectured that it functioned via a similar mechanism, and RIP-seq was performed to test this hypothesis [45]. Of the top 7 candidates with higher binding probability to UPF1 from the identified lncRNAs, LINC00963 exhibited the most notable difference in content between ECCs and ECSCs. The effect of the RNA pull-down experiment was less pronounced due to the large molecular weight of UPF1. Therefore, we performed a RIP assay to verify the binding with LINC00963. Interestingly, LINC00963, which stimulates aggressiveness in several cancers [46–49], was previously depicted as a potential pluripotency-associated gene in oral CSCs [24]. We found that LINC00963 contributed to oncogenic activities in EC. LINC00963 favored the expression of several stemness parameters to improve the self-renewal capacity and chemoresistance of ECSCs. Moreover, our results confirmed the specificity of UPF1 binding to LINC00963 in the cytoplasm, which enhanced the stability and expression of LINC00963. Notably, depletion of LINC00963 reinforced the suppression of aggressive features induced by UPF1 knockdown in vitro and in vivo.

Accumulated evidence indicates that lncRNAs serve as ceRNAs to crossregulate the expression of target genes by sponging miRNAs in stem cells [25–27]. For example, Linc-DYNC2H1-4 promotes CSC phenotypes by acting as a sponge of miR-145 in pancreatic CSCs [50]. In EC, Linc-RNA-RoR functioned as a sponge against mediation of the differentiation of ECSCs by miR-145 [51]. We discovered that LINC00963 and SOX2 shared the same miRNA response element for miR-508-5p, which affected ECSC behaviors. Another report demonstrated the suppressive effects of miR-508-5p on the differentiation of dental pulp stem cells via targeting of the protein GPNMB [28], which supports our finding.

The transcription factor SOX2 is a determinant in the maintenance of embryonic and induced pluripotent stem cells. Multiple studies demonstrated the oncogenic roles of SOX2 [29, 30]. SOX2 induces EGFR in a positive feedback manner in EC [31], which favors cell cycle progression via CDKN1A [32]. Particularly, SOX2 transcription was promoted by ALKBH5-induced mRNA demethylation, which maintained the stem-like state of ECSCs [52]. Our evidence nicely indicated that UPF1 dysregulation via the LINC00963/miR-508-5p axis may lead to an aberrant accumulation of SOX2 and supports the tumorigenicity potential of ECSCs, which broadens our understanding of SOX2-related regulatory mechanisms in ECSCs.

In conclusion, we demonstrated that UPF1 augmented the stability of LINC00963, which suppressed the negative regulation of miR-508-5p on the target gene SOX2 to promote ECSC properties. Inhibition of UPF1 may be used to lower the chances of CSCs being the tumor origin and may be beneficial for chemoprevention while sparing normal endometrial stem cells via the LINC00963/miR-508-5p/SOX2 pathway (Fig. 8J). Our results provide novel theoretical evidence for ECSC maintenance and new targets for EC treatment, especially highly refractory tumors.

## Supplementary information

Supplementary Legends

Supplementary Table S1

Supplementary Table S2

Supplementary Table S3

Supplementary Table S4

Supplementary Table S5

Supplementary Table S6

Supplementary Table S7

Supplementary Figure S1

Supplementary Figure S2

Supplementary Figure S3

Supplementary Figure S4

Supplementary Figure S5

Supplementary Figure S6

Supplementary Figure S7

Supplementary Figure S8

Supplementary Figure S9

Supplementary Figure S10

Original Data of WB

Author Contribution Statement

Checklist

## Data Availability

All western blots presented in figures were given in the section of Supplementary Materials which were named Original Data of WB. The data that support the findings of this study are available from the corresponding author upon reasonable request.
