# Supplementary material for: UPF1 contributes to the maintenance of endometrial cancer stem cell phenotype by stabilizing LINC00963
Source: Cell Death Dis. 2022 Mar 22;13(3):257. doi: 10.1038/s41419-022-04707-x (PMC8940903; doi:10.1038/s41419-022-04707-x)
Supplement: Supplementary file 1 — Supplementary Legends [file 41419_2022_4707_MOESM1_ESM.docx]

**Supplementary Fig. S1**  UPF1 is upregulated in EC tissues and differentially expressed in EC cell lines. (A) UPF1 expression levels in patients with different (A-i) tumor stages, (A-ii) races, (A-iii) ages, (A-iv) weights, (A-v) menopausal status, (A-vi) histological subtypes, and (A-vii) TP53 mutation statuses of EC in the TCGA cohort. Data are presented as the means ± SEM, **P<0.01, ***P<0.001. (B) UPF1 expression levels in EC cell lines and corresponding ECSCs assessed using Western blotting. The results are presented as the ratio of the integrated density values of UPF1 versus Tubulin and the graphs represent the alteration in relation to the Ishikawa cell line (protein of interest/Tubulin equal to 1). *P<0.05, **P<0.01 vs. Ishikawa.

**Supplementary Fig. S2**  The gate strategy for the selection of ECSCs. (A) Unstained control, (B) compensation control, and (C) isotype control were used to determine the specific gate.

**Supplementary Fig. S3**  Transfection efficiency of UPF1. The transfection efficiency of oe-UPF1 determined using (A) qRT-PCR and (B) Western blotting analyses. The transfection efficiency of sh-UPF1 determined using (C) qRT-PCR and (D) Western blotting analyses. The Western blotting results are presented as the ratio of the integrated density values of UPF1 versus Tubulin. The graphs represent the alteration in the oe-UPF1 group and the sh-UPF1 group relative to their respective control groups (protein of interest/Tubulin equal to 1). Data are presented as the means ± SEM (n = 3 per group), *P<0.05, ** P<0.01, *** P<0.001.

**Supplementary Fig. S4** Silencing UPF1 exerts tumor-suppressive effects on ECSCs. (A) Effect of UPF1 knockdown on SOX2, OCT4, and NANOG expression assessed using Western blotting. The results are presented as the ratio of the integrated density values of UPF1, SOX2, OCT4, and NANOG versus Tubulin. The graphs represent the alteration in relation to the sh-NC group (protein of interest/Tubulin equal to 1). (B) Effects of sh-UPF1 on self-renewal capacity assessed using serial sphere formation assay. (C) Effects of sh-UPF1 on carboplatin resistance assessed using the sphere formation assay. (D) Effects of sh-UPF1 on carboplatin resistance assessed by the CCK8 assay. (E) Effects of sh-UPF1 on proliferation assessed by the CCK8 assay. (F) Effects of sh-UPF1 on migration and invasion assessed using the Transwell assay. (G) Effects of sh-UPF1 on apoptosis assessed using flow cytometry analysis. (H) Effects of sh-UPF1 on cell cycle progression assessed using flow cytometry analysis. Data are presented as the means ± SEM (n=3, each group), **P<0.01, ***P<0.001 vs. sh-NC group. Scale bars, 50 μm.

**Supplementary Fig. S5**  Dose-response curves and corresponding IC50 values for ECCs and ECSCs exposed to a dose range of carboplatin.

**Supplementary Fig. S6**  Top 7 lncRNAs with higher binding probability to UPF1 and their expression levels in ECSCs. (A) Top 7 lncRNAs with higher binding probability to UPF1. (B) Expression of the top 7 lncRNAs with higher binding probability to UPF1 in ECCs and ECSCs detected using qRT-PCR. Data are presented as the means ± SEM (n=3, each group), *** P<0.001.

**Supplementary Fig. S7**  Transfection efficiency of LINC00963. (A) qRT-PCR analysis was used to determine the transfection efficiency of LINC00963-RNAi. Data are presented as the means ± SEM (n=3, each group), *** P<0.001. sh-LINC00963-1 was used in subsequent knock-down experiments according to the results. (B) qRT-PCR analysis was used to determine the transfection efficiency of LINC00963. Data are presented as the means ± SEM (n=3, each group), *** P<0.001 vs. oe-NC group, ###P<0.001 vs. sh-NC group.

**Supplementary Fig. S8**  Transfection efficiency of miR-508-5p. (A) Expression of miR-508-5p in ECCs and ECSCs detected using qRT-PCR. Data are presented as the means ± SEM (n=3, each group), **P<0.01. (B) qRT-PCR analysis was used to determine the transfection efficiency of miR-508-5p. Data are presented as the means ± SEM (n=3, each group), ***P<0.001 vs. Agomir-508-5p-NC group, ###P<0.001 vs. Antagomir-508-5p. (C) Effect of miR-508-5p overexpression or knockdown on LINC00963 expression using qRT-PCR. Data are presented as the means ± SEM (n=3, each group), **P<0.01, ***P<0.001 vs. Agomir-508-5p-NC group, ###P<0.001 vs. Antagomir-508-5p.

**Supplementary Fig. S9**  Expression of SOX2, OCT4, and NANOG in tumor xenografts assessed using immunohistochemistry. Scale bars, 50 μm.

­

**Supplementary Fig. S10** The survival curves of nude mice with xenografts (n=8, each group). P<0.05 for sh-UPF1 or sh- LINC00963 vs. sh-UPF1 + sh-LINC00963 group, P<0.01 for sh-UPF1 + sh-LINC00963 group vs. Control group. Using log-rank test for statistical analysis.

**Supplementary Table S1** Sequences of shRNA, plasmid, and RNA oligo/inhibitor template.

**Supplementary Table S2** Primers used for qRT-PCR.

**Supplementary Table S3** Primary antibodies used for the detection of protein expression.

**Supplementary Table S4** 332 LncRNA loci determined by RNA immunoprecipitation‑sequencing.

**Supplementary Table S5** Peaks of LINC00963 determined by RNA immunoprecipitation‑sequencing.

**Supplementary Table S6** Relationship of LINC00963 expression with clinical pathological parameters of tumor.

**Supplementary Table S7** Relationship of miR-508-5p expression with clinical pathological parameters of tumor.
