# Supplementary material for: UPF1 contributes to the maintenance of endometrial cancer stem cell phenotype by stabilizing LINC00963
Source: Cell Death Dis. 2022 Mar 22;13(3):257. doi: 10.1038/s41419-022-04707-x (PMC8940903; doi:10.1038/s41419-022-04707-x)
Supplement: Supplementary file 2 — Supplementary Table S1 [file 41419_2022_4707_MOESM2_ESM.docx]

**Supplementary Table S1**

Sequences of shRNA, plasmid, and RNA oligo/inhibitor template.

| Gene |  | Sequence(5'->3') |
| --- | --- | --- |
| NC | Sense | CACCGTTCTCCGAACGTGTCACGTCAAGAGATTACGTGACACGTTCGGAGAATTTTTTG |
|  | Antisense | GATCCAAAAAAGTTCTCCGAACGTGTCACGTAATCTCTTGACGTGACACGTTCGGAGAAC |
| sh-UPF1-1 | Sense | CCGGGCAAGGTATGGCGTCATCATTCTCGAGAATGATGACGCCATACCTTGCTTTTTG |
|  | Antisense | GATCCAAAAAGCAAGGTATGGCGTCATCATTCTCGAGAATGATGACGCCATACCTTGC |
| sh-UPF1-2 | Sense | CCGGCCAACCCGATAAACCGATGTTCTCGAGAACATCGGTTTATCGGGTTGGTTTTTG |
|  | Antisense | GATCCAAAAACCAACCCGATAAACCGATGTTCTCGAGAACATCGGTTTATCGGGTTGG |
| sh-UPF1-3 | Sense | CCGGGCCTACCAGTACCAGAACATACTCGAG TATGTTCTGGTACTGGTAGGC TTTTTG |
|  | Antisense | GATCCAAAAAGCCTACCAGTACCAGAACATACTCGAGTATGTTCTGGTACTGTAGGC |
| LINC00963-RNAi-1 | Sense | CCGGGAGCTTTCCAGCCTGTGCTAACTCGAGTTAGCACAGGCTGGAAAGCTTTTTTG |
|  | Antisense | AATTCAAAAAGAGCTTTCCAGCCTGTGCTAACTCGAGTTAGCACAGGCTGGAAAGCTC |
| LINC00963-RNAi-2 | Sense | CACCGGCAAGTGCTTTCAACTCTCTCTCGAGAGAGAGTTGAAAGCACTTGCCTTTTTG |
|  | Antisense | GATCCAAAAAGGCAAGTGCTTTCAACTCTCTCTCGAGAGAGAGTTGAAAGCACTTGCC |
| LINC00963-RNAi-3 | Sense | CACCGCCACCCGGAATTACAATTCTCTCGAGAGAATTGTAATTCCGGGTGGCTTTTTG |
|  | Antisense | GATCCAAAAAGCCACCCGGAATTACAATTCTCTCGAGAGAATTGTAATTCCGGGTGGC |
| miR-Stable Negative Control | Sense | UUCUCCGAACGUGUCACGUTT |
|  | Antisense | ACGUGACACGUUCGGAGAATT |
| Agomir-508-5p | Sense | UACUCCAGAGGGCGUCACUCAUG |
|  | Antisense | UGAGUGACGCCCUCUGGAGUAUU |
| miR-Inhibitor Negative Control |  | CAGUACUUUUGUGUAGUACAA |
| Antagomir-508-5p |  | CAUGAGUGACGCCCUCUGGAGUA |
