# Supplementary material for: UPF1 contributes to the maintenance of endometrial cancer stem cell phenotype by stabilizing LINC00963
Source: Cell Death Dis. 2022 Mar 22;13(3):257. doi: 10.1038/s41419-022-04707-x (PMC8940903; doi:10.1038/s41419-022-04707-x)
Supplement: Supplementary file 3 — Supplementary Table S2 [file 41419_2022_4707_MOESM3_ESM.docx]

**Supplementary Table S2**

Primers used for qRT-PCR.

| Gene | Sequence (5'->3') |
| --- | --- |
| UPF1 | F: GAAGCTGGTCAACACTATCAAC |
|  | R: GTCATGGGTCTGGAAGTACATG |
| LINC00963 | F: TGGAGATGGAAGGAGGATGCTCAC |
| miR-508-5p | R: CAGAAGTGTTCAGGCGTGGACTC  RT: GTCGTATCCAGTGCAGGGTCCGAGGTATTCGCACTGGATACGACCATGAG  F: CGTACTCCAGAGGGCGTCA  R: AGTGCAGGGTCCGAGGTATT |
| SOX2 | F: ACGCTCATGAAGAAGGATAAGT |
|  | R: GAGCTGGTCATGGAGTTGTAC |
| GAPDH | F: GGAGCGAGATCCCTCCAAAAT  R: GGCTGTTGTCATACTTCTCATGG |
| U6 | RT: GTCGTATCCAGTGCAGGGTCCGAGGTATTCGCACTGGATACGACAAAATA  F: AGAGAAGATTAGCATGGCCCCTG  R: ATCCAGTGCAGGGTCCGAGG |

F: Forward; R: Reverse.
