# Supplementary material for: UPF1 contributes to the maintenance of endometrial cancer stem cell phenotype by stabilizing LINC00963
Source: Cell Death Dis. 2022 Mar 22;13(3):257. doi: 10.1038/s41419-022-04707-x (PMC8940903; doi:10.1038/s41419-022-04707-x)
Supplement: Supplementary file 4 — Supplementary Table S3 [file 41419_2022_4707_MOESM4_ESM.docx]

**Supplementary Table S3**

Primary antibodies used for the detection of protein expression.

| Name | Manufacturer | Dilution ratio:  Western blotting, Immunofluorescence |
| --- | --- | --- |
| UPF1 | Abcam, Cambridge, UK | 1:25000, 1:500 |
| SOX2 | Abcam, Cambridge, UK | 1:2000 |
| OCT4 | Abcam, Cambridge, UK | 1:8000 |
| NANOG | Abcam, Cambridge, UK | 1:8000 |
