# Supplementary material for: UPF1 contributes to the maintenance of endometrial cancer stem cell phenotype by stabilizing LINC00963
Source: Cell Death Dis. 2022 Mar 22;13(3):257. doi: 10.1038/s41419-022-04707-x (PMC8940903; doi:10.1038/s41419-022-04707-x)
Supplement: Supplementary file 5 — Supplementary Table S4 [file 41419_2022_4707_MOESM5_ESM.docx]

**Supplementary Table S4**

332 LncRNA loci determined by RNA immunoprecipitation‑sequencing.

| Gene | Start | End |
| --- | --- | --- |
| RP11-34P13.7 | 129054 | 129223 |
| RP11-34P13.13 | 141473 | 143011 |
| RP11-34P13.13 | 146385 | 149707 |
| RP11-34P13.9 | 160445 | 160690 |
| RP11-34P13.9 | 161313 | 161525 |
| RP11-34P13.13 | 164262 | 164791 |
| RP11-34P13.13 | 168609 | 168767 |
| RP11-34P13.13 | 169048 | 169264 |
| RP11-34P13.13 | 172556 | 172688 |
| RP11-34P13.13 | 173752 | 173862 |
| AP006222.2 | 287516 | 287921 |
| AP006222.2 | 289265 | 289370 |
| AP006222.2 | 297344 | 297502 |
| RP5-857K21.15 | 357382 | 357586 |
| RP5-857K21.15 | 358048 | 358183 |
| RP4-669L17.2 | 358856 | 358957 |
| RP5-857K21.15 | 359344 | 359681 |
| RP4-669L17.2 | 360056 | 360168 |
| RP4-669L17.10 | 365170 | 366120 |
| RP4-669L17.10 | 368250 | 368450 |
| RP4-669L17.10 | 373143 | 373323 |
| RP4-669L17.10 | 379768 | 379972 |
| RP4-669L17.10 | 476530 | 476945 |
| RP4-669L17.10 | 484831 | 485208 |
| RP4-669L17.10 | 494381 | 494898 |
| RP4-669L17.10 | 495276 | 495476 |
| RP4-669L17.10 | 496263 | 496665 |
| RP4-669L17.10 | 497108 | 497299 |
| RP4-669L17.10 | 498046 | 498305 |
| RP4-669L17.10 | 498398 | 499369 |
| RP5-857K21.4 | 585988 | 586358 |
| RP5-857K21.4 | 586820 | 586955 |
| RP5-857K21.2 | 587628 | 587729 |
| RP5-857K21.4 | 588116 | 588453 |
| RP5-857K21.4 | 594234 | 594768 |
| RP5-857K21.4 | 595082 | 595217 |
| RP5-857K21.4 | 597298 | 597498 |
| RP5-857K21.4 | 601397 | 601577 |
| RP5-857K21.4 | 607954 | 608056 |
| RP5-857K21.4 | 627376 | 627823 |
| RP5-857K21.4 | 627960 | 628223 |
| RP5-857K21.4 | 701935 | 702340 |
| RP5-857K21.4 | 703684 | 703789 |
| RP5-857K21.4 | 711341 | 711922 |
| LINC01128 | 853790 | 853914 |
| CTD-2012J19.3 | 1610518 | 1610642 |
| RP11-161I6.2 | 1883523 | 1884062 |
| KBTBD11-OT1 | 1958398 | 1958547 |
| LINC00102 | 2612987 | 2613696 |
| LINC00102 | 2614716 | 2614915 |
| LINC00102 | 2614991 | 2615347 |
| RP11-706O15.1 | 3817534 | 3817778 |
| RP11-706O15.1 | 3821680 | 3822285 |
| RP11-706O15.1 | 3822373 | 3822472 |
| RP11-706O15.1 | 3824233 | 3824598 |
| RP11-706O15.1 | 3828505 | 3829392 |
| RP11-706O15.1 | 3843340 | 3843539 |
| RP11-706O15.1 | 3843690 | 3843857 |
| RP11-706O15.3 | 3853009 | 3854927 |
| RP11-706O15.3 | 3860789 | 3860996 |
| RP11-706O15.3 | 3864397 | 3865142 |
| RP11-706O15.3 | 3866921 | 3867806 |
| RP11-706O15.3 | 3881764 | 3882317 |
| RP11-706O15.5 | 3891437 | 3893351 |
| RP11-706O15.5 | 3899215 | 3899422 |
| RP11-706O15.5 | 3905279 | 3906232 |
| RP11-706O15.5 | 3920193 | 3920746 |
| RP11-706O15.7 | 3929868 | 3930442 |
| RP11-706O15.7 | 3931018 | 3931242 |
| RP11-428J1.5 | 5042962 | 5043186 |
| RP11-428J1.5 | 5043237 | 5043386 |
| AC092171.4 | 5476453 | 5476652 |
| RP11-717F1.2 | 5553706 | 5553838 |
| RP11-717F1.2 | 5565962 | 5566086 |
| RP11-717F1.2 | 5579669 | 5579808 |
| RP11-717F1.2 | 5582227 | 5584101 |
| RP11-717F1.2 | 5584240 | 5586369 |
| RP11-717F1.2 | 5588136 | 5591960 |
| CH507-24F1.2 | 6232565 | 6232833 |
| CH507-24F1.2 | 6234982 | 6235192 |
| CH507-24F1.2 | 6235988 | 6236160 |
| CH507-24F1.2 | 6267134 | 6267317 |
| CH507-145C22.1 | 6320871 | 6320974 |
| CH507-145C22.1 | 6321163 | 6321717 |
| CH507-145C22.1 | 6354263 | 6354522 |
| CH507-145C22.1 | 6356687 | 6356986 |
| CH507-145C22.1 | 6358554 | 6360415 |
| CH507-152C13.6 | 6550748 | 6551450 |
| CH507-152C13.6 | 6552766 | 6552930 |
| CH507-152C13.6 | 6553769 | 6553955 |
| CH507-154B10.1 | 6630181 | 6632437 |
| CH507-154B10.1 | 6634357 | 6634656 |
| CH507-154B10.1 | 6637078 | 6637202 |
| CH507-154B10.1 | 6659518 | 6659740 |
| CH507-145C22.3 | 6667303 | 6668254 |
| CH507-145C22.3 | 6670521 | 6670695 |
| CH507-254M2.3 | 6721811 | 6721985 |
| CH507-254M2.1 | 7080035 | 7080289 |
| CH507-254M2.1 | 7081003 | 7081213 |
| CH507-254M2.1 | 7083362 | 7083742 |
| CH507-254M2.1 | 7086869 | 7087043 |
| CH507-254M2.2 | 7461813 | 7462067 |
| CH507-254M2.2 | 7462781 | 7462991 |
| CH507-254M2.2 | 7465140 | 7465784 |
| CH507-254M2.2 | 7467022 | 7468996 |
| CH507-338C24.1 | 7669446 | 7673264 |
| CH507-338C24.1 | 7675413 | 7675512 |
| RP11-20D14.6 | 8788256 | 8788530 |
| FAM66A | 12362018 | 12362562 |
| FAM66A | 12368238 | 12368615 |
| FAM66A | 12387740 | 12387865 |
| RP11-419I17.1 | 12537078 | 12539065 |
| LINC00958 | 12979534 | 12980536 |
| LINC00958 | 12980684 | 12980852 |
| AC005546.2 | 13131570 | 13131895 |
| AF127936.7 | 14823067 | 14823191 |
| AF127936.9 | 14823392 | 14823491 |
| AF127936.9 | 14823667 | 14823766 |
| DUXAP8 | 15784958 | 15785057 |
| DUXAP8 | 15787171 | 15787282 |
| DUXAP8 | 15788551 | 15788699 |
| DUXAP8 | 15788819 | 15788931 |
| DUXAP8 | 15790660 | 15790798 |
| DUXAP8 | 15791009 | 15791152 |
| DUXAP8 | 15791627 | 15791814 |
| DUXAP8 | 15818492 | 15819165 |
| LRRC75A-AS1 | 16439034 | 16440253 |
| RP11-138I1.3 | 16440478 | 16440657 |
| RP11-138I1.3 | 16440827 | 16440952 |
| MIR99AHG | 16607668 | 16607842 |
| MIR99AHG | 16608243 | 16608342 |
| MIR99AHG | 16608993 | 16609267 |
| LINC01296 | 19335351 | 19335462 |
| LINC01296 | 19337579 | 19337686 |
| RP11-94H6.1 | 19747178 | 19747318 |
| LINC00641 | 21201203 | 21201384 |
| LINC01239 | 22682469 | 22682713 |
| LINC01239 | 22723308 | 22723430 |
| LINC01239 | 22820862 | 22820961 |
| LINC01224 | 23400707 | 23400856 |
| RP11-255H23.4 | 23817623 | 23818397 |
| RP11-255H23.4 | 23818548 | 23819122 |
| RP11-255H23.4 | 23823739 | 23823906 |
| SPATA13 | 24297587 | 24297735 |
| SPATA13 | 24306422 | 24306596 |
| U91328.19 | 25998027 | 25998276 |
| U91328.19 | 25998577 | 25998676 |
| RP4-760C5.3 | 26010304 | 26010531 |
| AP000230.1 | 26170895 | 26171058 |
| RP4-752I6.1 | 27457197 | 27457338 |
| RP4-752I6.1 | 27459295 | 27459582 |
| RP11-296K13.4 | 29403725 | 29404105 |
| DKFZP434L187 | 30211591 | 30211740 |
| LINC00941 | 30800210 | 30800559 |
| LINC00941 | 30801700 | 30801874 |
| RP3-430N8.11 | 30978519 | 30978744 |
| RP11-84A19.4 | 31921356 | 31921841 |
| RP4-534N18.2 | 31933019 | 31933210 |
| RP4-534N18.2 | 31933649 | 31933975 |
| AC021224.1 | 32412181 | 32412687 |
| LINC00997 | 32760903 | 32761077 |
| RP11-11N9.4 | 33044691 | 33044855 |
| LINC00665 | 36313191 | 36313815 |
| LINC00665 | 36314116 | 36316070 |
| LINC00665 | 36320250 | 36320399 |
| LINC00665 | 36320550 | 36320689 |
| LINC00665 | 36324936 | 36327225 |
| LINC00665 | 36330252 | 36330492 |
| LINC00665 | 36331446 | 36331695 |
| EBLN3 | 37080858 | 37081049 |
| LINC00839 | 42477335 | 42477442 |
| LINC00839 | 42486978 | 42487077 |
| LINC00839 | 42492179 | 42492303 |
| LINC00839 | 42494739 | 42494963 |
| RP11-242D8.1 | 43162239 | 43162463 |
| RP5-1198O20.4 | 44030442 | 44030557 |
| RP11-616K22.2 | 44387319 | 44387488 |
| RP11-616K22.2 | 44389965 | 44390451 |
| EIF3J-AS1 | 44536167 | 44536291 |
| RP11-398E10.1 | 44763531 | 44763705 |
| RP11-398E10.1 | 44764856 | 44764980 |
| RP11-398E10.1 | 44765106 | 44765626 |
| SNHG15 | 44983614 | 44983945 |
| AL133493.2 | 45602440 | 45602539 |
| CRHR1-IT1 | 45620609 | 45621152 |
| CRHR1-IT1 | 45621907 | 45622031 |
| CRHR1-IT1 | 45629978 | 45630152 |
| RP11-474P2.4 | 46384232 | 46384668 |
| RP11-474P2.4 | 46386865 | 46386991 |
| RP11-96H19.1 | 46387746 | 46387845 |
| ZNF674-AS1 | 46547665 | 46547823 |
| RP11-156P1.3 | 47056206 | 47056355 |
| RP11-156P1.3 | 47056531 | 47056708 |
| RP11-430B1.2 | 52204964 | 52205537 |
| TRAM2-AS1 | 52577606 | 52577905 |
| TRAM2-AS1 | 52577981 | 52578130 |
| TRAM2-AS1 | 52582862 | 52583086 |
| TRAM2-AS1 | 52583662 | 52583886 |
| USP46-AS1 | 52661541 | 52661665 |
| RP11-44F14.2 | 53384548 | 53384697 |
| LINC01602 | 57983665 | 57983964 |
| RP11-111F5.4 | 62376522 | 62376640 |
| RP11-111F5.8 | 62387170 | 62387510 |
| RP11-111F5.8 | 62391105 | 62392229 |
| RP11-111F5.8 | 62392555 | 62394004 |
| SNHG1 | 62854275 | 62854466 |
| SNHG1 | 62854691 | 62855236 |
| LINC01278 | 63432363 | 63432491 |
| LINC01278 | 63560831 | 63561071 |
| RP11-301G19.1 | 67888195 | 67888319 |
| PCBP1-AS1 | 70030347 | 70030446 |
| PCBP1-AS1 | 70031347 | 70031496 |
| PCBP1-AS1 | 70031532 | 70031821 |
| PCBP1-AS1 | 70032132 | 70032255 |
| PCBP1-AS1 | 70045258 | 70046796 |
| PCBP1-AS1 | 70048686 | 70048957 |
| PCBP1-AS1 | 70049044 | 70049493 |
| PCBP1-AS1 | 70049519 | 70050268 |
| PCBP1-AS1 | 70050599 | 70051305 |
| PCBP1-AS1 | 70053730 | 70054180 |
| PCBP1-AS1 | 70055616 | 70055715 |
| PCBP1-AS1 | 70055730 | 70055910 |
| PCBP1-AS1 | 70056191 | 70056601 |
| PCBP1-AS1 | 70085915 | 70086325 |
| LINC00511 | 72403921 | 72404070 |
| LINC00511 | 72404246 | 72404399 |
| LINC00511 | 72404421 | 72404574 |
| LINC00511 | 72422784 | 72423070 |
| RP11-57A1.1 | 72425938 | 72426182 |
| LINC00511 | 72427971 | 72428852 |
| LINC00511 | 72445013 | 72445119 |
| LINC00511 | 72470058 | 72470366 |
| LINC00511 | 72592602 | 72592726 |
| RP11-169F17.1 | 73158921 | 73159595 |
| RP11-169F17.1 | 73250317 | 73250466 |
| RP11-169F17.1 | 73255597 | 73255918 |
| RP11-169F17.1 | 73264239 | 73264338 |
| JPX | 73944331 | 73944946 |
| JPX | 73946998 | 73947430 |
| RP13-216E22.4 | 73949355 | 73949454 |
| JPX | 73994839 | 73994961 |
| JPX | 73998953 | 74000491 |
| JPX | 74004269 | 74004493 |
| AP001372.2 | 74496648 | 74496947 |
| LINC01291 | 74929363 | 74929512 |
| LINC00960 | 75672390 | 75672489 |
| LINC00960 | 75674412 | 75675837 |
| LINC00960 | 75678907 | 75679006 |
| SNHG16 | 76557765 | 76560433 |
| SNHG16 | 76561287 | 76561751 |
| RP11-488C13.5 | 76786109 | 76786283 |
| SNHG20 | 77088722 | 77088847 |
| SNHG20 | 77094218 | 77094368 |
| PKIA-AS1 | 78533891 | 78535721 |
| PKIA-AS1 | 78535849 | 78535973 |
| PKIA-AS1 | 78556498 | 78556599 |
| RP11-379B8.1 | 81845559 | 81845783 |
| CTD-2284J15.1 | 86343081 | 86343314 |
| LUCAT1 | 91311020 | 91312419 |
| ARRDC3-AS1 | 91380348 | 91381020 |
| LINC01578 | 92882806 | 92883022 |
| LINC01578 | 92883584 | 92883861 |
| LINC01578 | 92887755 | 92888314 |
| LINC01578 | 92897723 | 92898022 |
| LINC01578 | 92899098 | 92899247 |
| LINC01578 | 92899348 | 92899497 |
| RP11-185E12.2 | 93743002 | 93743500 |
| RP11-1070N10.5 | 95642092 | 95642191 |
| RP11-485F13.1 | 98864835 | 98864984 |
| RP11-12G12.7 | 100086380 | 100086554 |
| OLMALINC | 100373614 | 100373763 |
| OLMALINC | 100373789 | 100374012 |
| OLMALINC | 100381215 | 100383368 |
| RPARP-AS1 | 102449830 | 102450266 |
| RPARP-AS1 | 102450292 | 102451147 |
| RPARP-AS1 | 102451385 | 102453539 |
| RPARP-AS1 | 102453839 | 102456213 |
| KB-1254G8.1 | 102855377 | 102856075 |
| RP11-152P17.3 | 105826569 | 105826740 |
| RP11-152P17.3 | 105833764 | 105834013 |
| LINC01593 | 108049561 | 108049785 |
| LINC01593 | 108052633 | 108052755 |
| EPB41L4A-AS1 | 112161702 | 112162276 |
| LINC01234 | 113744726 | 113745025 |
| LINC01234 | 113745728 | 113745852 |
| LINC01234 | 113746176 | 113746327 |
| LINC01234 | 113746351 | 113746595 |
| LINC01234 | 113751013 | 113752188 |
| LINC01234 | 113753430 | 113753563 |
| LINC01234 | 113767074 | 113767209 |
| SAMD12-AS1 | 118621000 | 118621186 |
| SAMD12-AS1 | 118621534 | 118621655 |
| RP11-4K16.2 | 119215110 | 119215627 |
| RP11-4K16.2 | 119221093 | 119221407 |
| RP11-4K16.2 | 119223283 | 119223387 |
| RP11-4K16.2 | 119223803 | 119223999 |
| RP11-33B1.3 | 119456792 | 119457277 |
| MIR100HG | 122090902 | 122091108 |
| RP11-486O12.2 | 123584530 | 123585115 |
| PVT1 | 127998199 | 127998359 |
| PVT1 | 128009589 | 128009706 |
| PVT1 | 128099694 | 128099887 |
| PVT1 | 128100979 | 128101153 |
| LINC00963 | 129490818 | 129491128 |
| LINC00963 | 129493065 | 129493595 |
| LINC00963 | 129495444 | 129495705 |
| LINC00963 | 129496922 | 129497073 |
| LINC00963 | 129501989 | 129503139 |
| LINC00963 | 129503397 | 129503622 |
| LINC00963 | 129503722 | 129503846 |
| RP11-989F5.3 | 130810605 | 130810958 |
| RP11-989F5.3 | 130812133 | 130812622 |
| C10orf91 | 132447736 | 132447860 |
| C10orf91 | 132448611 | 132448835 |
| C10orf91 | 132448986 | 132449160 |
| C10orf91 | 132449228 | 132449335 |
| RP11-472G23.10 | 150040546 | 150041173 |
| RP11-472G23.10 | 150041780 | 150042033 |
| RP11-54C4.3 | 151770616 | 151770772 |
| GAS5 | 173865242 | 173865547 |
| GAS5 | 173865817 | 173866796 |
| GAS5 | 173866990 | 173867815 |
| HAGLROS | 176178424 | 176179008 |
| CTC-338M12.9 | 181281399 | 181281498 |
| CTC-338M12.9 | 181283341 | 181283490 |
| LINC00467 | 211382844 | 211382975 |
| LINC00467 | 211390563 | 211390670 |
| LINC00467 | 211391826 | 211391976 |
| LINC00467 | 211398035 | 211398638 |
| LINC00467 | 211399257 | 211399437 |
| LINC00467 | 211433043 | 211433892 |
| FLVCR1-AS1 | 212857790 | 212858088 |
