# Supplementary material for: UPF1 contributes to the maintenance of endometrial cancer stem cell phenotype by stabilizing LINC00963
Source: Cell Death Dis. 2022 Mar 22;13(3):257. doi: 10.1038/s41419-022-04707-x (PMC8940903; doi:10.1038/s41419-022-04707-x)
Supplement: Supplementary file 6 — Supplementary Table S5 [file 41419_2022_4707_MOESM6_ESM.docx]

| Chr | Start | End | Length | Strand | Fold enrichment | P value |
| --- | --- | --- | --- | --- | --- | --- |
| 9 | 129490818 | 129491128 | 311 | + | 29 | 1.53E-10 |
| 9 | 129493065 | 129493595 | 531 | + | 29 | 1.53E-05 |
| 9 | 129495444 | 129495705 | 262 | + | 12 | 0.000890839 |
| 9 | 129496922 | 129497073 | 152 | + | 12.5 | 0.00063012 |
| 9 | 129501989 | 129503139 | 1151 | + | 9 | 0.006884027 |
| 9 | 129503397 | 129503622 | 226 | + | 29 | 1.53E-05 |
| 9 | 129503722 | 129503846 | 125 | + | 9.5 | 0.004918387 |

**Supplementary Table S5**

Peaks of LINC00963 determined by RNA immunoprecipitation‑sequencing.
