# Supplementary material for: UPF1 contributes to the maintenance of endometrial cancer stem cell phenotype by stabilizing LINC00963
Source: Cell Death Dis. 2022 Mar 22;13(3):257. doi: 10.1038/s41419-022-04707-x (PMC8940903; doi:10.1038/s41419-022-04707-x)
Supplement: Supplementary file 7 — Supplementary Table S6 [file 41419_2022_4707_MOESM7_ESM.docx]

**Supplementary Table S6**

Relationship of LINC00963 expression with clinical pathological parameters of tumor.

| Clinical pathological parameters |  | N = 58 | LINC00963  Mean ± SEM | *P* |
| --- | --- | --- | --- | --- |
| Age | < 60 | 39 | 4.959 ± 0.982 | 0.275 |
|  | ≥ 60 | 19 | 3.289 ± 0.792 |  |
| Clinical stage | I + II | 47 | 2.486 ± 0.329 | **0.001** |
|  | III + IV | 11 | 12.641 ± 2.186 |  |
| Differentiation | High | 30 | 5.502 ± 1.220 | 0.109 |
|  | Low&Middle | 28 | 3.244 ± 0.640 |  |
| Invasion depth | < 1/2Muscle layer | 46 | 2.982 ± 0.480 | **0.015** |
|  | ≥1/2Muscle layer | 12 | 9.894 ±2.381 |  |
| Lymph node metastasis | Negative | 51 | 3.076 ± 0.433 | **0.014** |
|  | Positive | 7 | 14.149 ± 3.265 |  |
