# Supplementary material for: UPF1 contributes to the maintenance of endometrial cancer stem cell phenotype by stabilizing LINC00963
Source: Cell Death Dis. 2022 Mar 22;13(3):257. doi: 10.1038/s41419-022-04707-x (PMC8940903; doi:10.1038/s41419-022-04707-x)
Supplement: Supplementary file 8 — Supplementary Table S7 [file 41419_2022_4707_MOESM8_ESM.docx]

**Supplementary Table S7**

Relationship of miR-508-5p expression with clinical pathological parameters of tumor.

| Clinical pathological parameters |  | N = 58 | miR-508-5p  Mean ± SEM | *P* |
| --- | --- | --- | --- | --- |
| Age | < 60 | 39 | 0.551 ± 0.096 | 0.641 |
|  | ≥ 60 | 19 | 0.569 ± 0.123 |  |
| Clinical stage | I + II | 47 | 0.666 ± 0.086 | **<0.001** |
|  | III + IV | 11 | 0.090 ± 0.034 |  |
| Differentiation | High | 30 | 0.461 ± 0.081 | 0.191 |
|  | Low&Middle | 28 | 0.660 ± 0.130 |  |
| Invasion depth | < 1/2 Muscle layer | 46 | 0.654 ± 0.089 | **<0.001** |
|  | ≥1/2 Muscle layer | 12 | 0.186 ± 0.060 |  |
| Lymph node metastasis | Negative | 51 | 0.620 ± 0.082 | **<0.001** |
|  | Positive | 7 | 0.097 ± 0.055 |  |
