# Supplementary figures and images for: UPF1 contributes to the maintenance of endometrial cancer stem cell phenotype by stabilizing LINC00963
Source: Cell Death Dis. 2022 Mar 22;13(3):257. doi: 10.1038/s41419-022-04707-x (PMC8940903; doi:10.1038/s41419-022-04707-x)

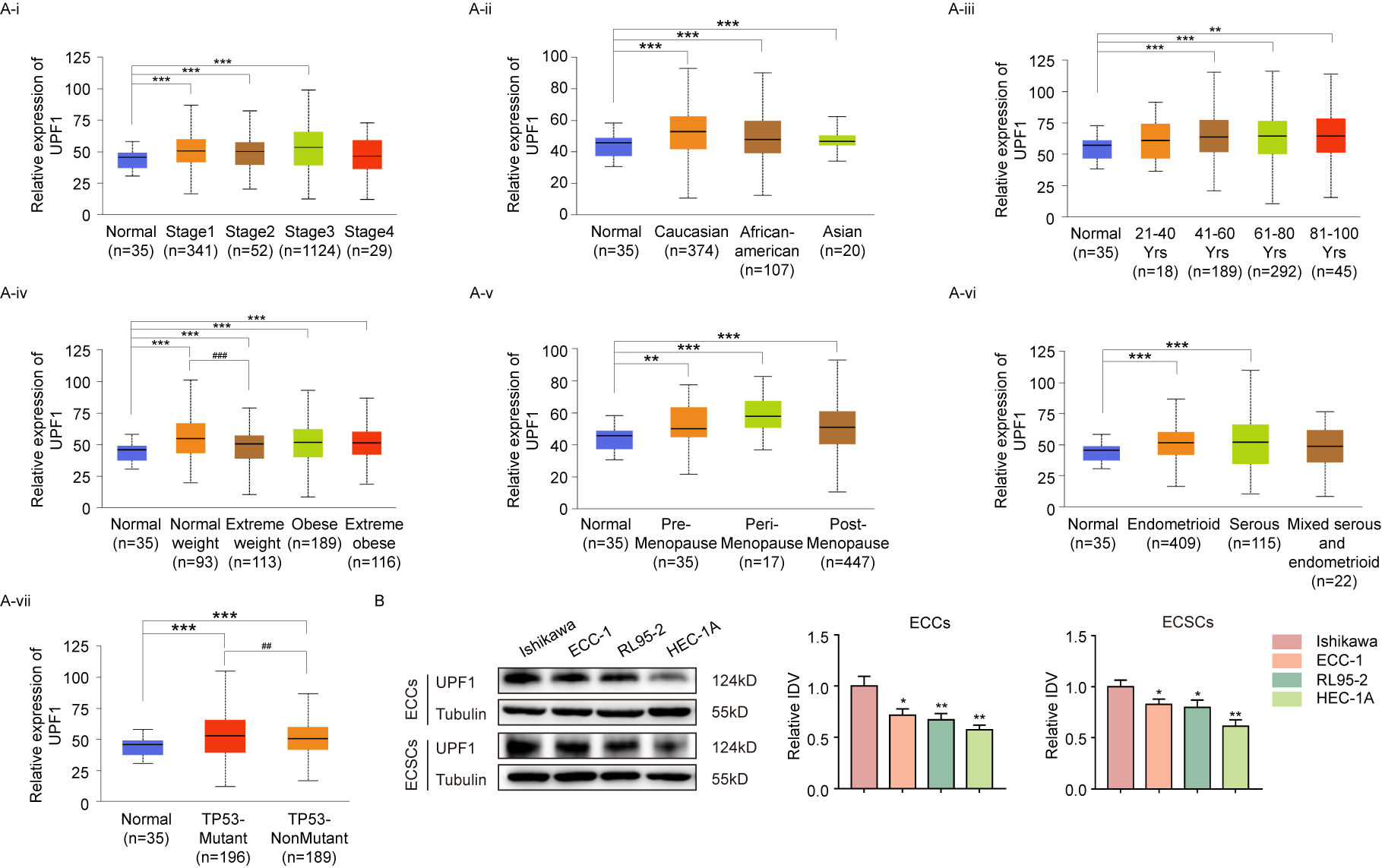

Supplement: Supplementary file 9 — Supplementary Figure S1 [file 41419_2022_4707_MOESM9_ESM.tif]

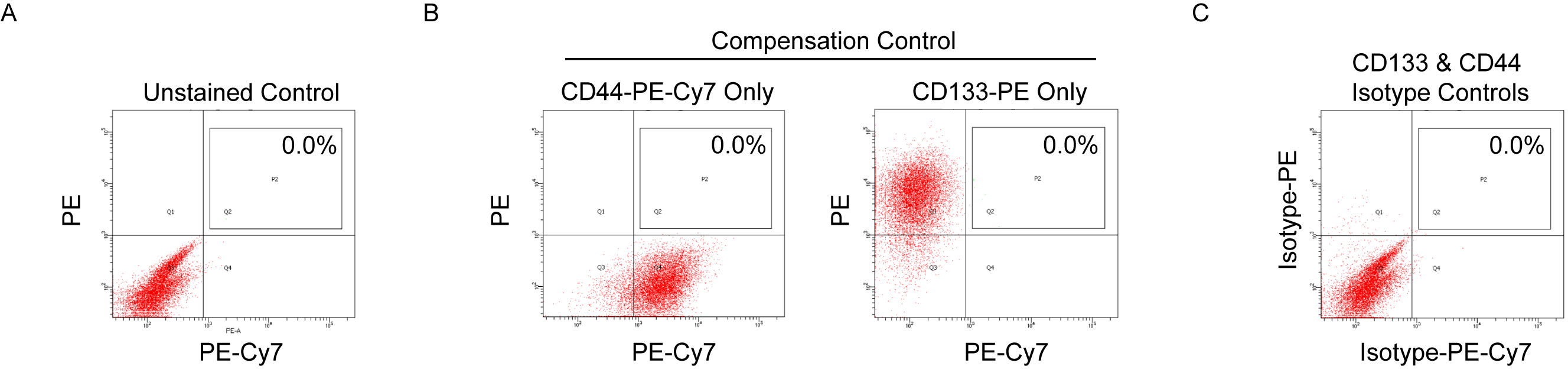

Supplement: Supplementary file 10 — Supplementary Figure S2 [file 41419_2022_4707_MOESM10_ESM.tif]

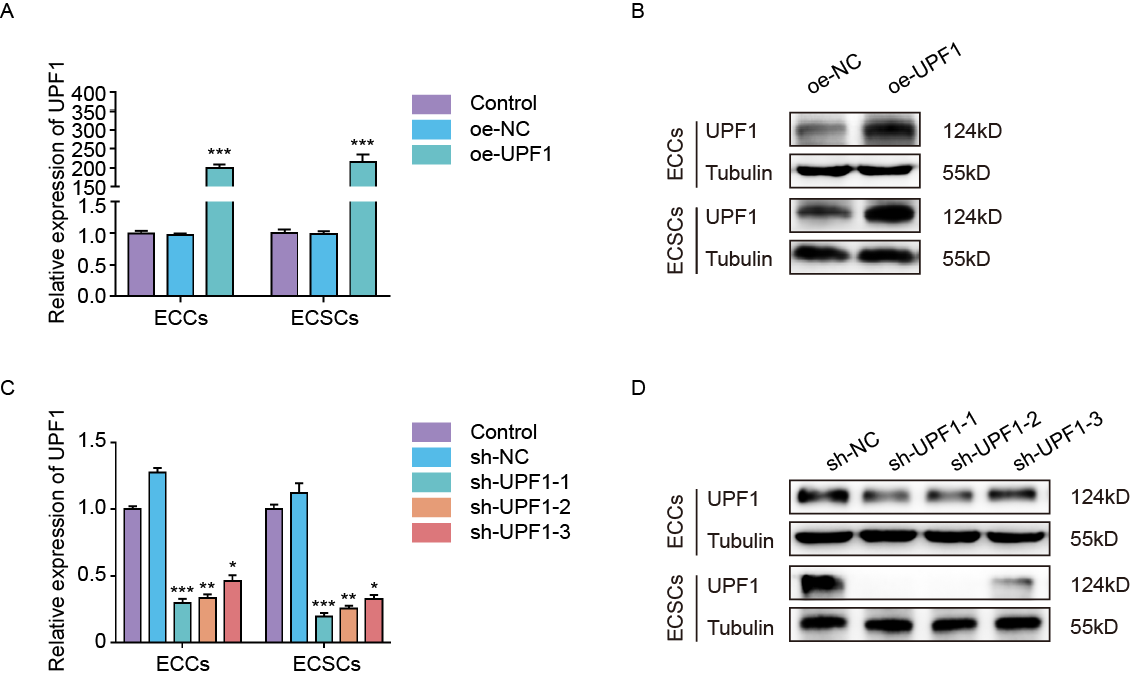

Supplement: Supplementary file 11 — Supplementary Figure S3 [file 41419_2022_4707_MOESM11_ESM.tif]

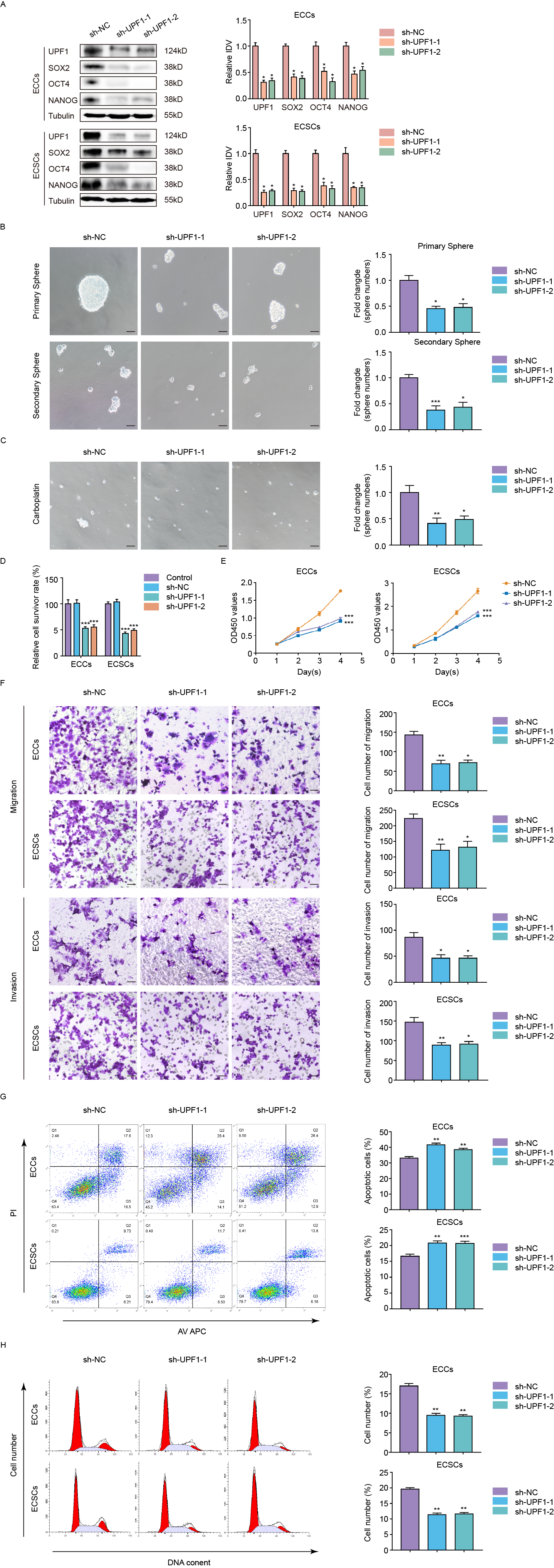

Supplement: Supplementary file 12 — Supplementary Figure S4 [file 41419_2022_4707_MOESM12_ESM.tif]

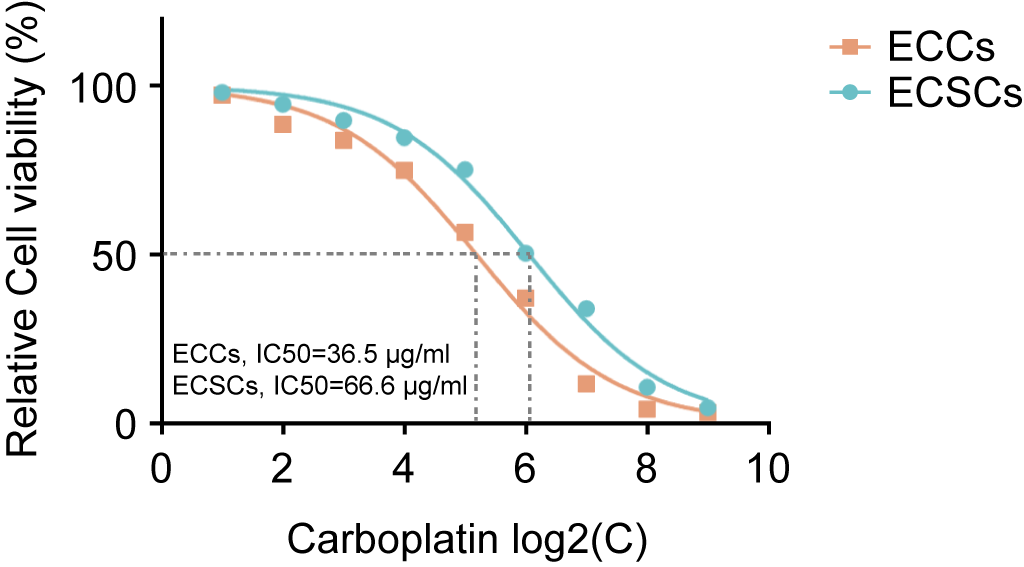

Supplement: Supplementary file 13 — Supplementary Figure S5 [file 41419_2022_4707_MOESM13_ESM.tif]

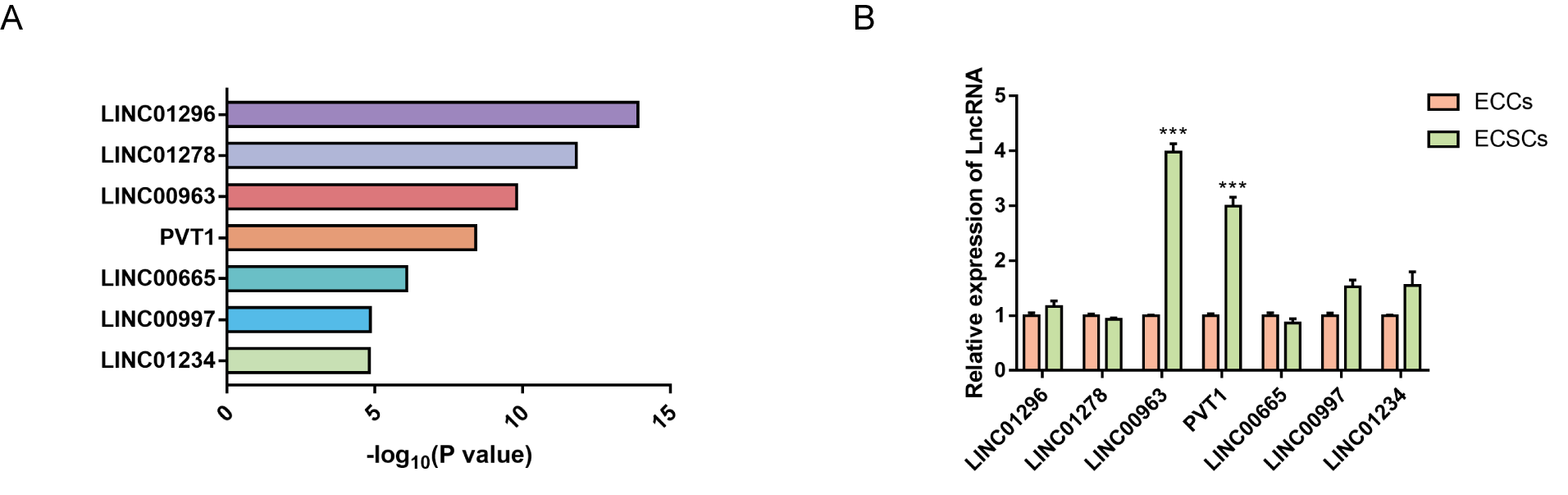

Supplement: Supplementary file 14 — Supplementary Figure S6 [file 41419_2022_4707_MOESM14_ESM.tif]

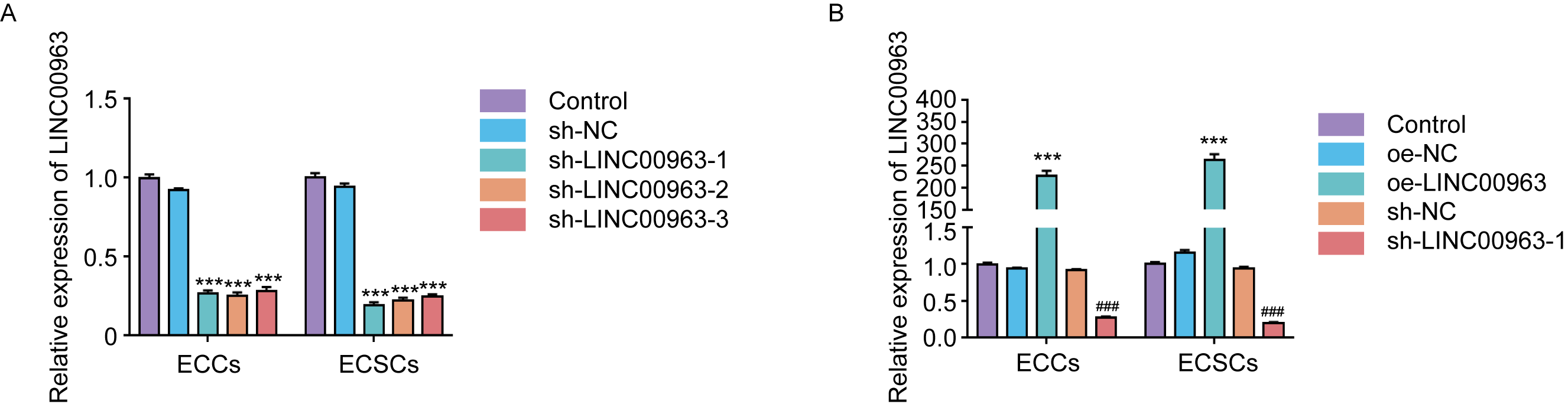

Supplement: Supplementary file 15 — Supplementary Figure S7 [file 41419_2022_4707_MOESM15_ESM.tif]

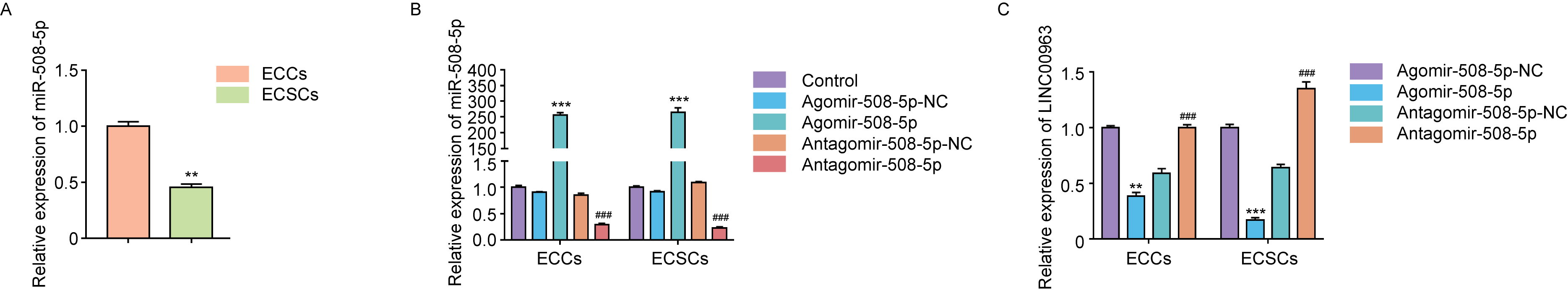

Supplement: Supplementary file 16 — Supplementary Figure S8 [file 41419_2022_4707_MOESM16_ESM.tif]

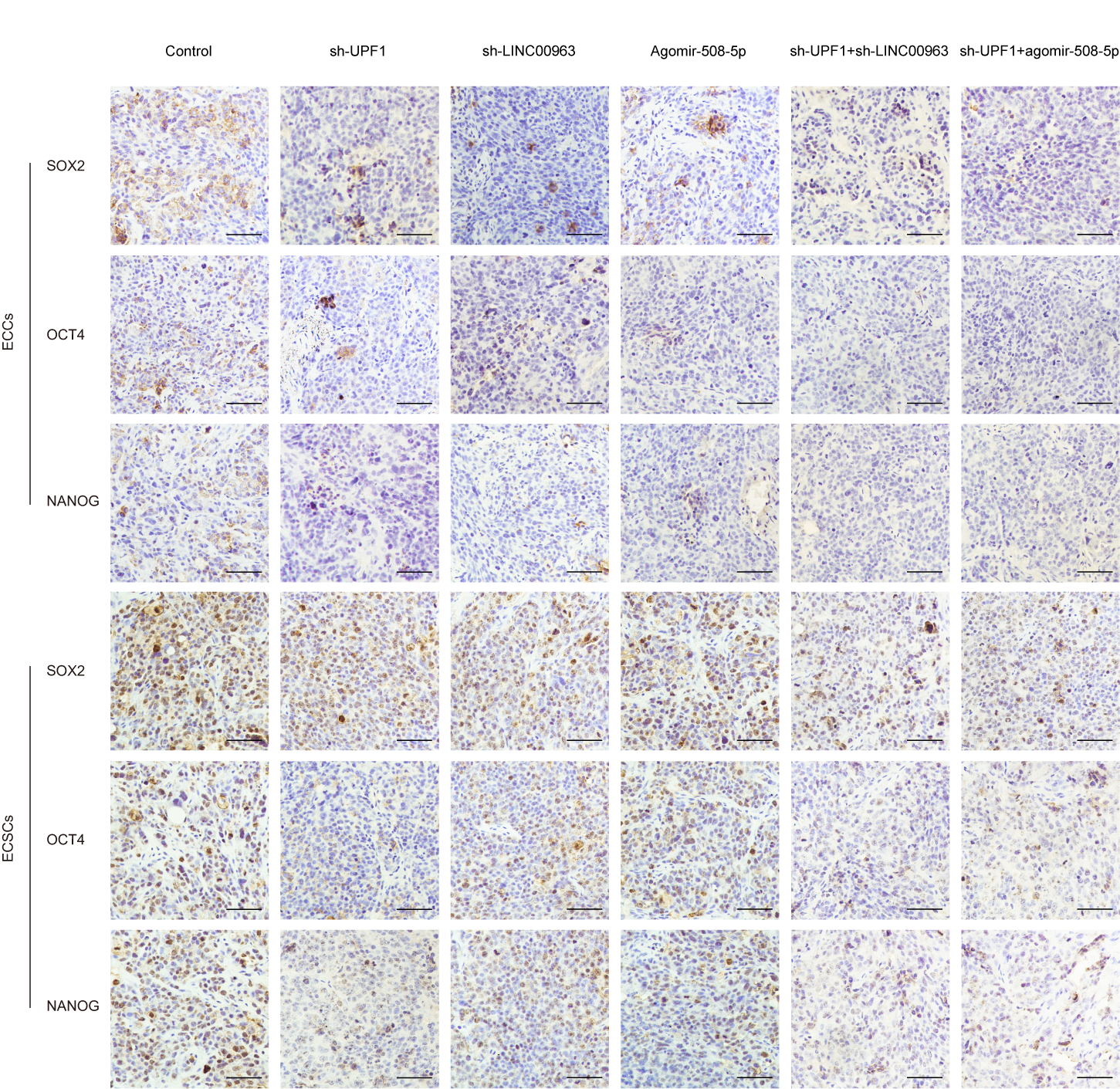

Supplement: Supplementary file 17 — Supplementary Figure S9 [file 41419_2022_4707_MOESM17_ESM.tif]

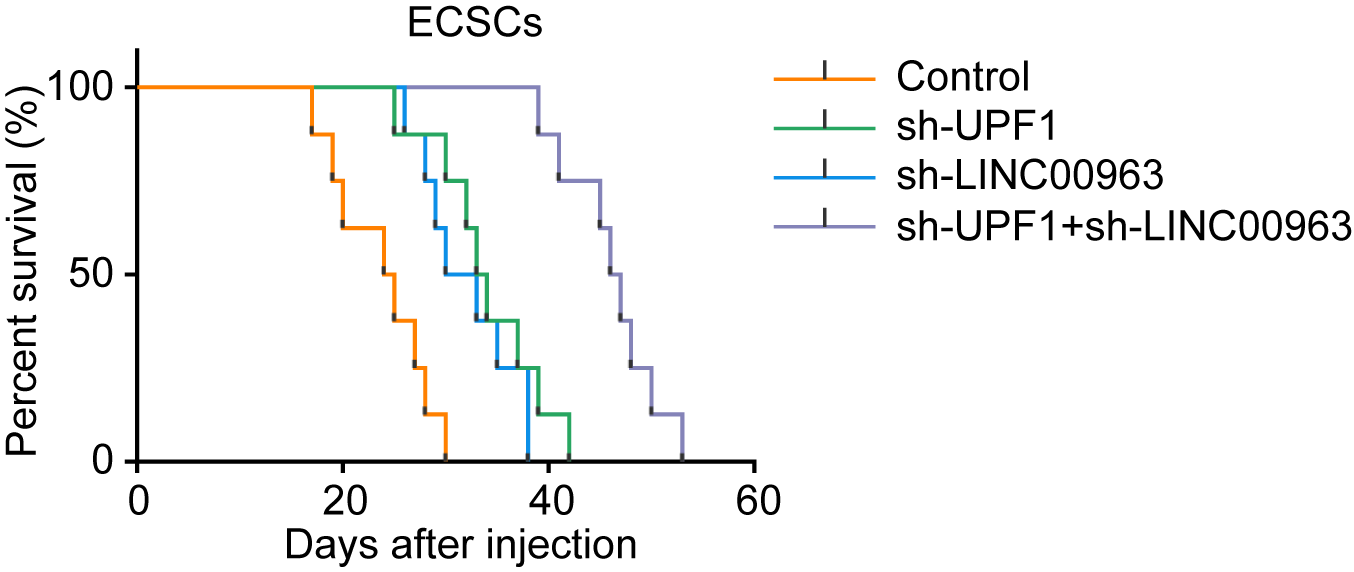

Supplement: Supplementary file 18 — Supplementary Figure S10 [file 41419_2022_4707_MOESM18_ESM.tif]
