# Supplementary material for: UPF1 contributes to the maintenance of endometrial cancer stem cell phenotype by stabilizing LINC00963
Source: Cell Death Dis. 2022 Mar 22;13(3):257. doi: 10.1038/s41419-022-04707-x (PMC8940903; doi:10.1038/s41419-022-04707-x)
Supplement: Supplementary file 19 — Original Data of WB [file 41419_2022_4707_MOESM19_ESM.pdf]

Fig. 1C

UPF1 (lane 1-6)

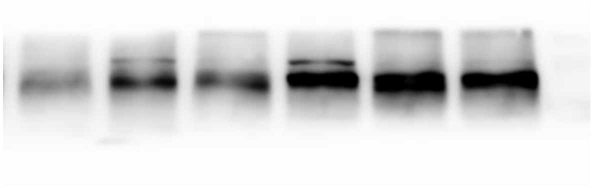

124kD

Tubulin (lane 1-6)

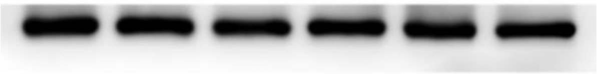

55kD

Fig. 1F

UPF1 (lane 3-5)

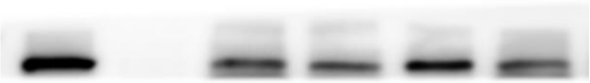

124kD

SOX2 (lane 3-5)

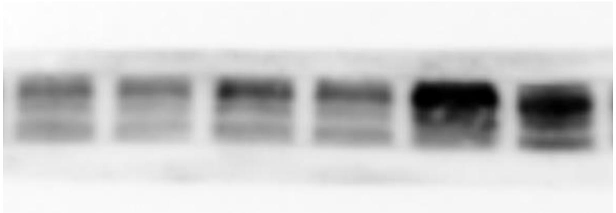

38kD

OCT4 (lane 3-5)

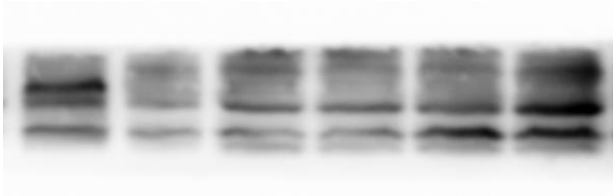

38kD

NANOG (lane 3-5)

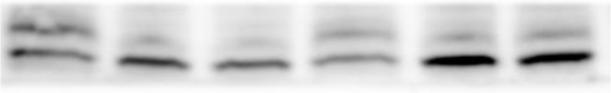

38kD

Tubulin (lane 3-5)

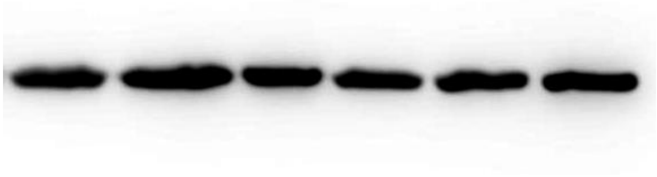

55kD

Fig. 1G

UPF1 (lane 1-4)

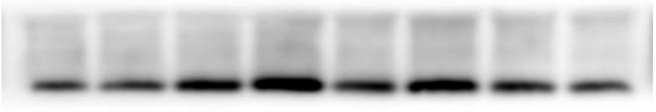

124kD

SOX2 (lane 4-7)

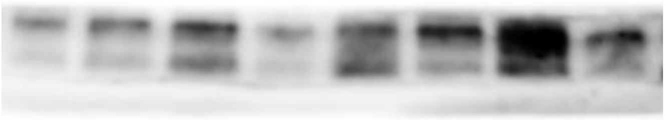

38kD

OCT4 (lane 1-4)

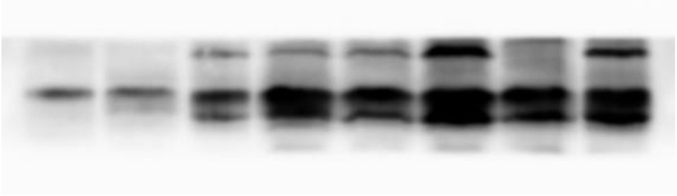

38kD

NANOG (lane 2-5)

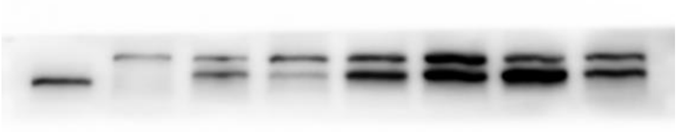

38kD

Tubulin (lane 1-4)

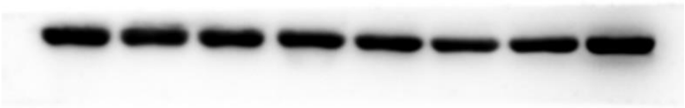

55kD

Fig. 1H

ECCs

UPF1 (lane 1-4)

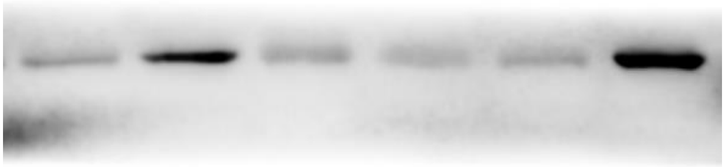

124kD

SOX2 (lane 3-6)

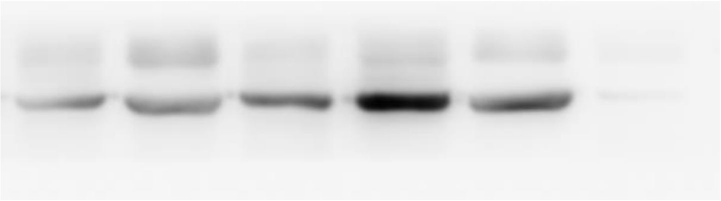

38kD

OCT4 (lane 1-4)

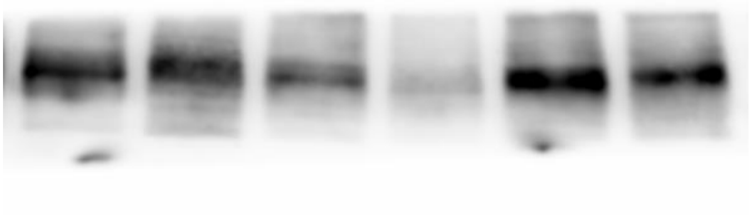

38kD

NANOG (lane 1-4)

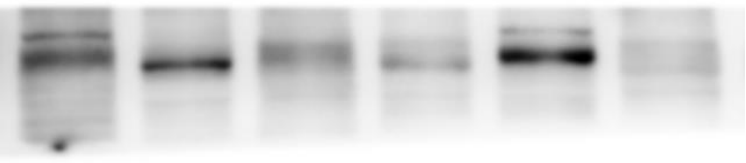

38kD

Tubulin (lane 1-4)

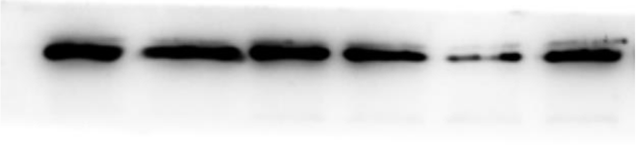

55kD

ECSCs

UPF1 (lane 3-6)

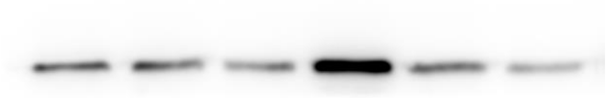

124kD

SOX2 (lane 1-4)

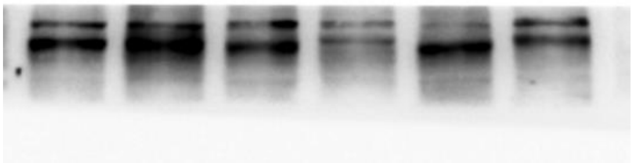

38kD

OCT4 (lane 1-4)

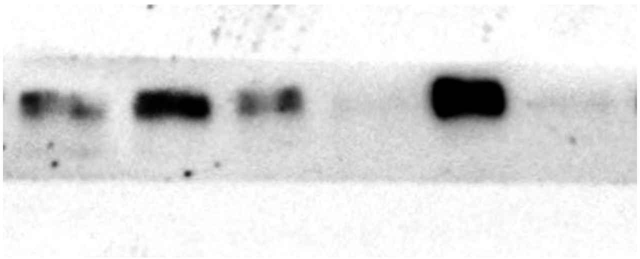

38kD

NANOG (lane 3-6)

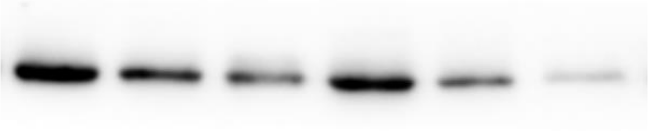

38kD

Tubulin (lane 3-6)

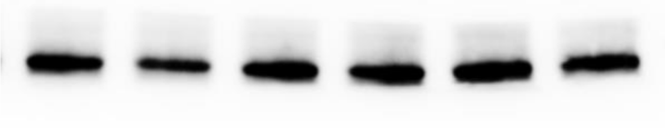

55kD

Fig. 4B

ECCs

SOX2 (lane 1-4)

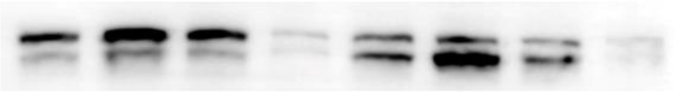

38kD

OCT4 (lane 1-4)

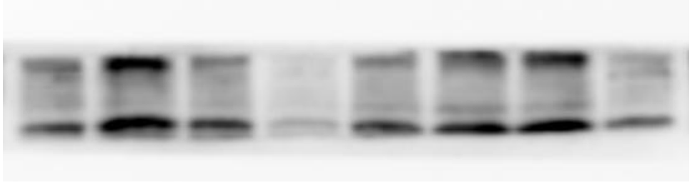

38kD

NANOG (lane 5-8)

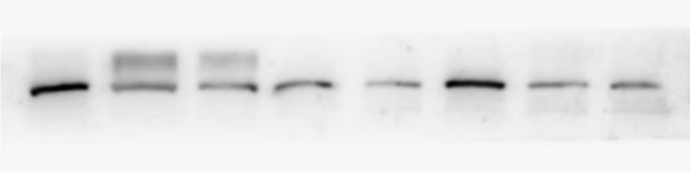

38kD

Tubulin (lane 5-8)

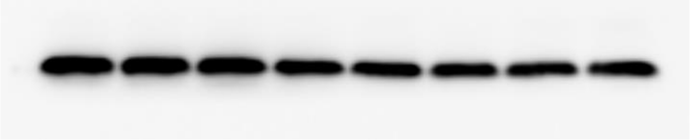

55kD

ECSCs

SOX2 (lane 1-4)

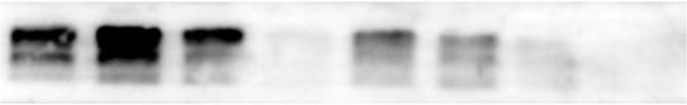

38kD

OCT4 (lane 5-8)

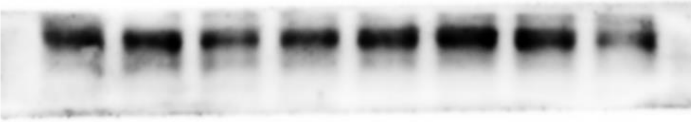

38kD

NANOG (lane 1-4)

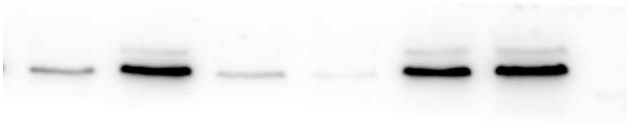

38kD

Tubulin (lane 5-8)

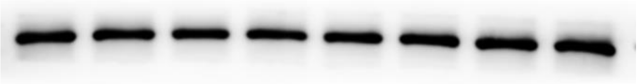

55kD

Fig. 5B

ECCs

SOX2 (lane 1-4)

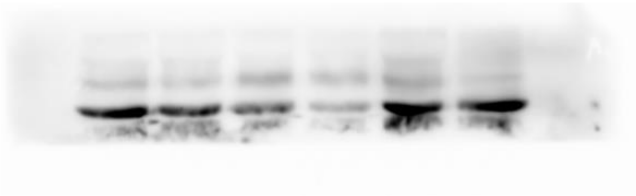

38kD

OCT4 (lane 5-8)

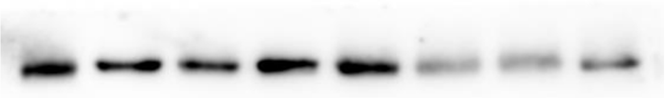

38kD

NANOG (lane 1-4)

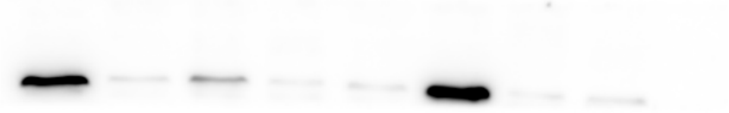

38kD

Tubulin (lane 5-8)

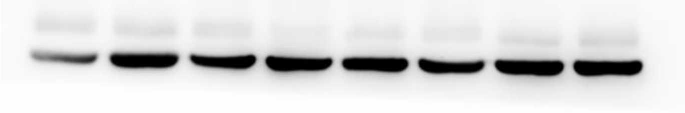

55kD

ECSCs

SOX2 (lane 1-4)

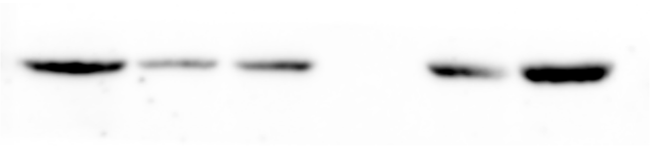

38kD

OCT4 (lane 3-6)

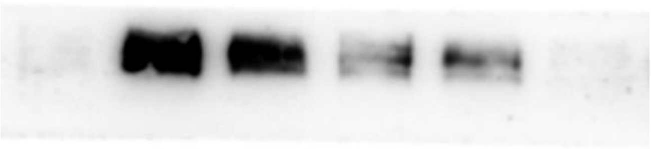

38kD

NANOG (lane 3-6)

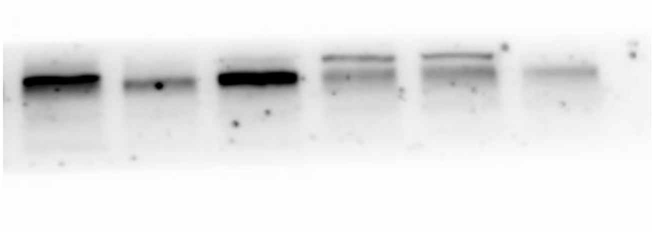

38kD

Tubulin (lane 1-4)

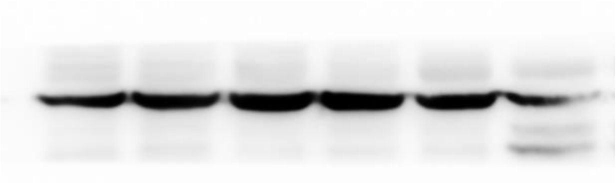

55kD

Fig. 6E

ECCs

SOX2 (lane 3-6)

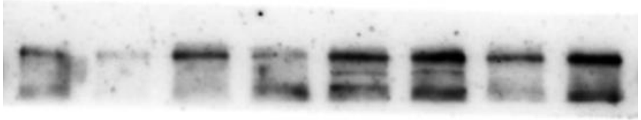

38kD

OCT4 (lane 1-4)

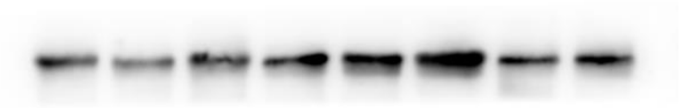

38kD

NANOG (lane 1-4)

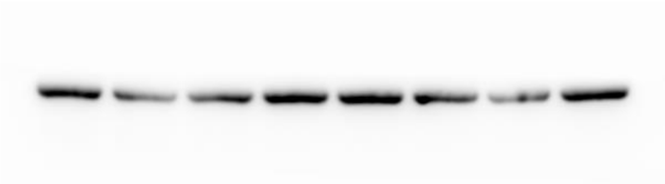

38kD

Tubulin (lane 1-4)

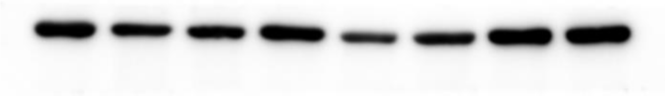

55kD

ECSCs

SOX2 (lane 2-5)

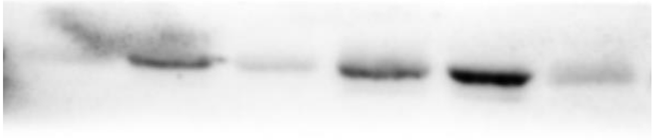

38kD

OCT4 (lane 1-4)

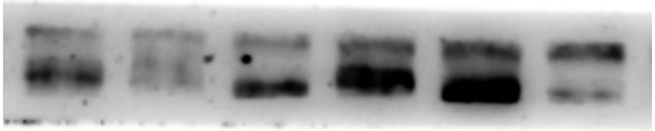

38kD

NANOG (lane 2-5)

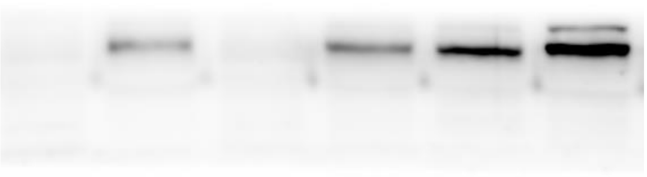

38kD

Tubulin (lane 2-5)

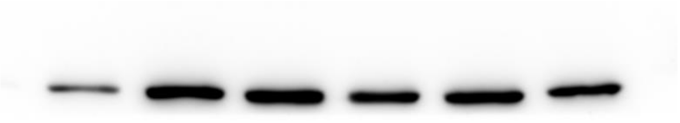

55kD

Fig. 7B

ECCs

SOX2 (lane 5-8)

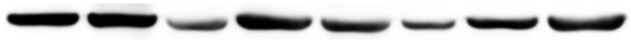

38kD

OCT4 (lane 1-4)

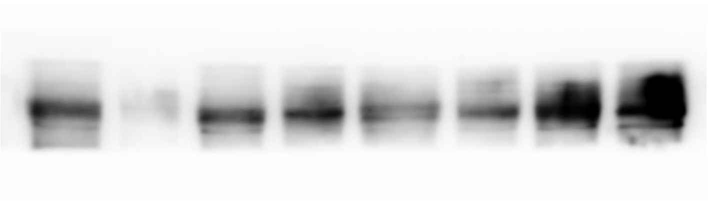

38kD

NANOG (lane 1-4)

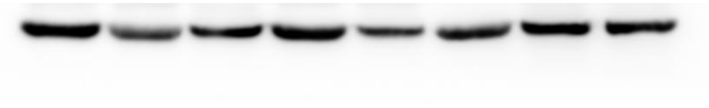

38kD

Tubulin (lane 1-4)

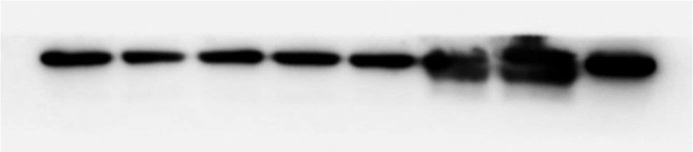

55kD

ECSCs

SOX2 (lane 1-4)

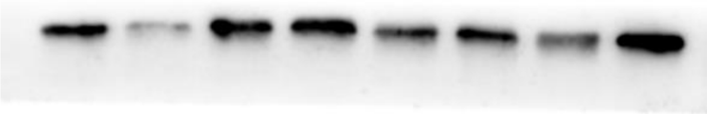

38kD

OCT4 (lane 5-8)

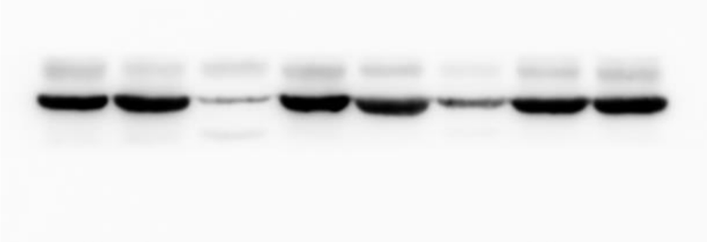

38kD

NANOG (lane 3-6)

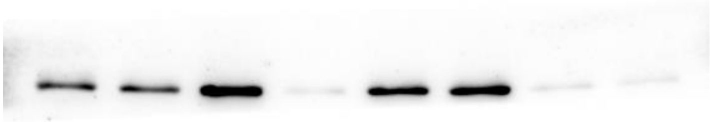

38kD

Tubulin (lane 5-8)

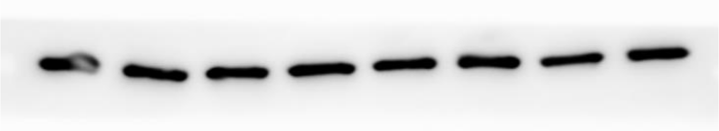

55kD

Fig. 8G

ECCs

SOX2 (lane 2-7)

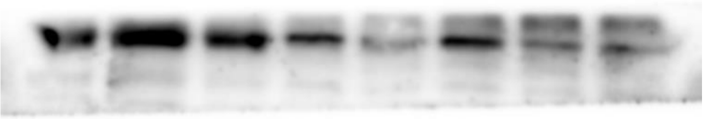

38kD

OCT4 (lane 2-7)

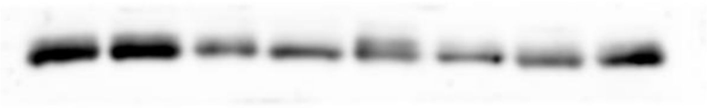

38kD

NANOG (lane 1-6)

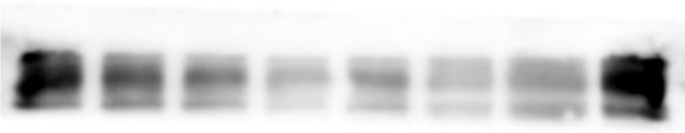

38kD

Tubulin (lane 2-7)

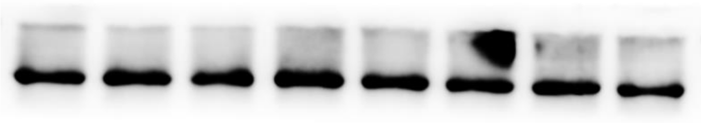

55kD

ECSCs

SOX2 (lane 2-7)

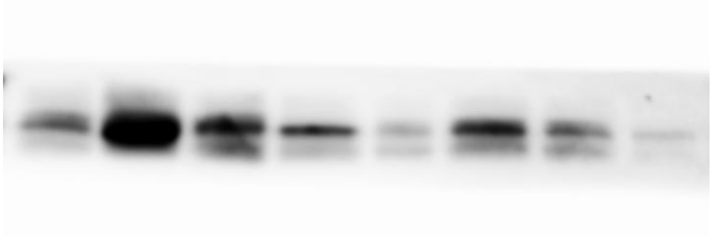

38kD

OCT4 (lane 1-6)

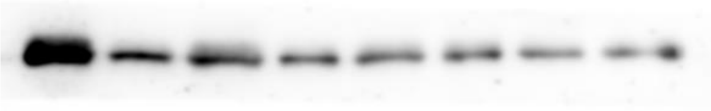

38kD

NANOG (lane 1-6)

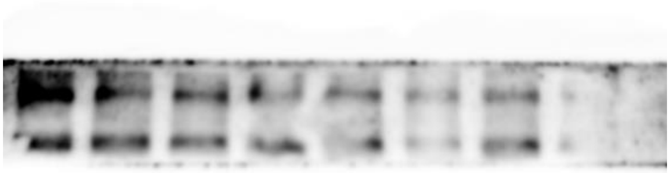

38kD

Tubulin (lane 1-6)

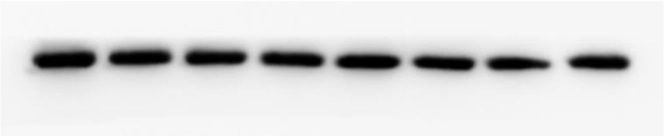

55kD

Supplementary Fig. S1B

ECCs

UPF1 (lane 2-5)

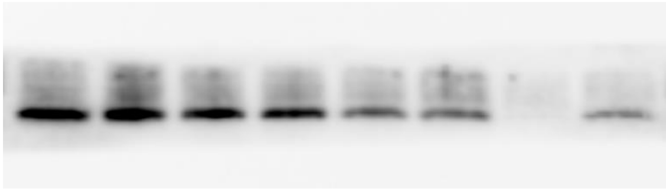

124kD

Tubulin (lane 2-5)

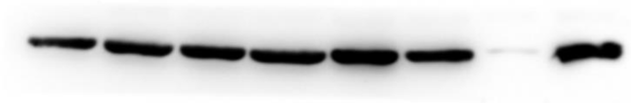

55kD

ECSCs

UPF1 (lane 1-4)

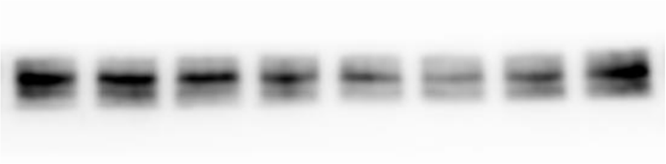

124kD

Tubulin (lane 1-4)

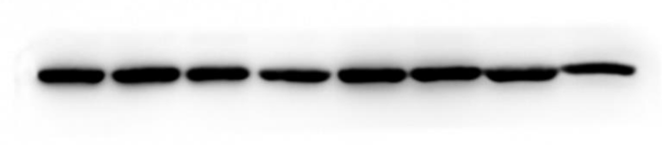

55kD

Supplementary Fig. S3B

ECCs

UPF1 (lane 6-7)

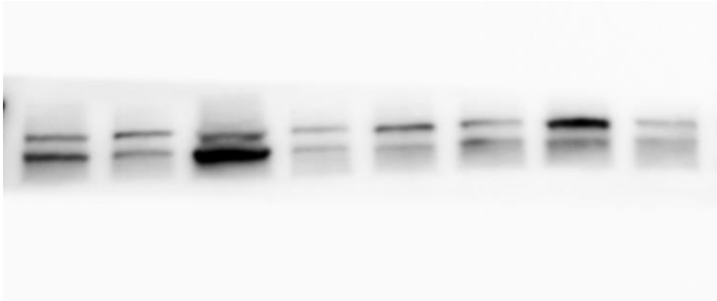

124kD

Tubulin (lane 6-7)

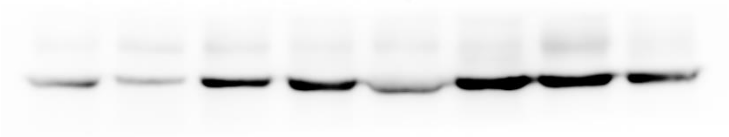

55kD

ECSCs

UPF1 (lane 6-7)

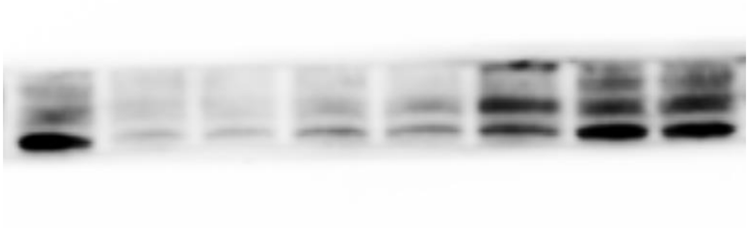

124kD

Tubulin (lane 6-7)

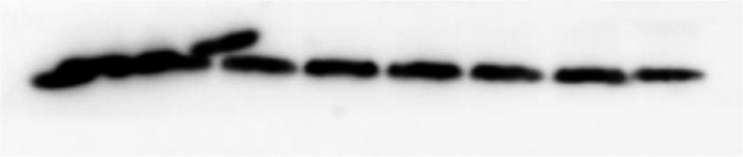

55kD

Supplementary Fig. S3D

ECCs

UPF1 (lane 1-4)

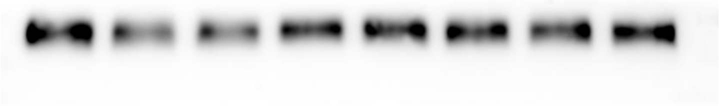

124kD

Tubulin (lane 1-4)

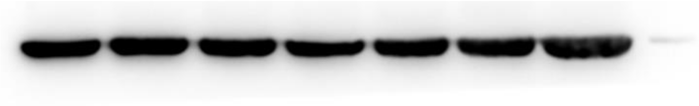

ECSCs

UPF1 (lane 1-4)

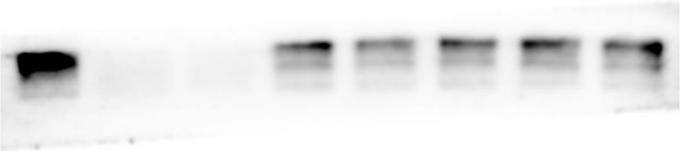

124kD

Tubulin (lane 1-4)

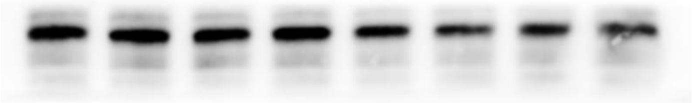

55kD

Supplementary Fig. S4A

ECCs

UPF1 (lane 2-4)

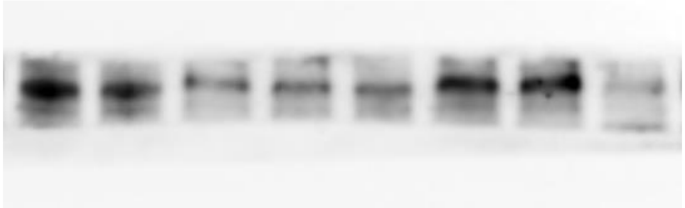

124kD

SOX2 (lane 2-4)

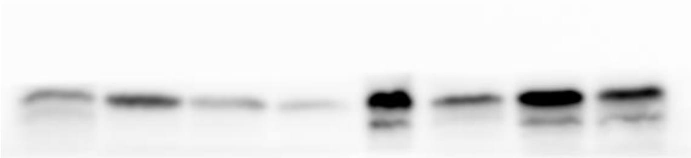

38kD

OCT4 (lane 1-3)

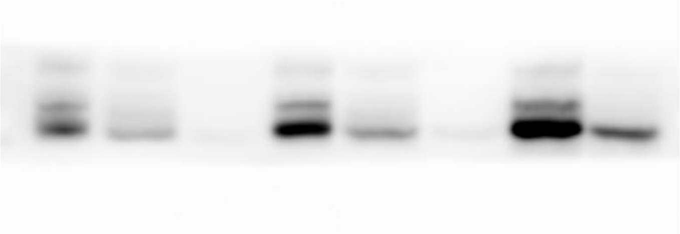

38kD

NANOG (lane 6-8)

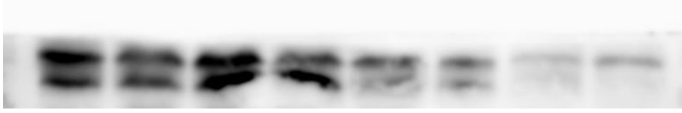

38kD

Tubulin (lane 1-3)

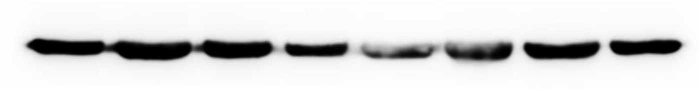

55kD

ECSCs

UPF1 (lane 2-4)

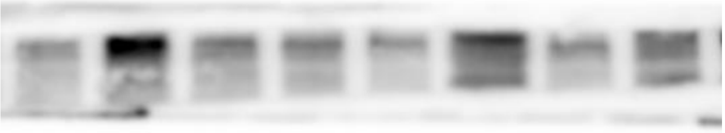

124kD

SOX2 (lane 3-5)

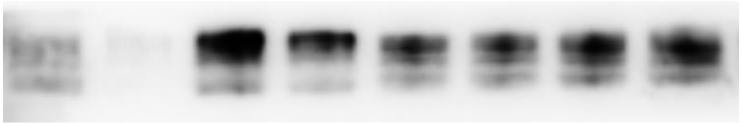

38kD

OCT4 (lane 4-6)

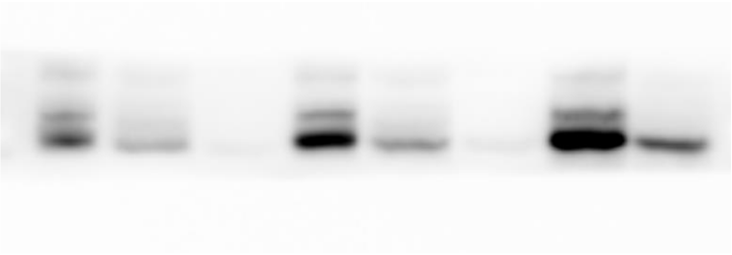

38kD

NANOG (lane 3-5)

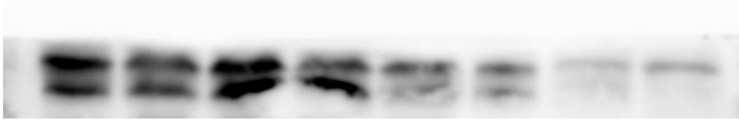

38kD

Tubulin (lane 2-4)

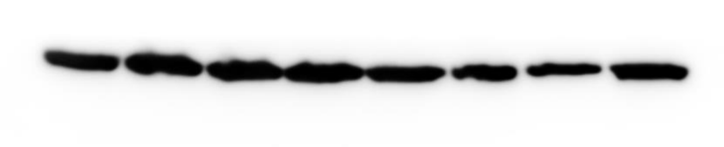

55kD
