# Supplementary material for: UPF1 contributes to the maintenance of endometrial cancer stem cell phenotype by stabilizing LINC00963
Source: Cell Death Dis. 2022 Mar 22;13(3):257. doi: 10.1038/s41419-022-04707-x (PMC8940903; doi:10.1038/s41419-022-04707-x)
Supplement: Supplementary file 20 — Author Contribution Statement [file 41419_2022_4707_MOESM20_ESM.docx]

Conception and design: X.M. Development and methodology: H.C., J.M., F.K. Acquisition of data: H.C., J.M., F.K., C.W. Analysis and interpretation of data: H.C., F.K., N.S., C.W. Administrative, technical, or material support: X.M., J.M., F.K., N.S. Study supervision: X.M. Writing, review, and/or revision of the manuscript: All authors. Final approval: All authors.
